# Signaling ammonium across membranes through an ammonium sensor histidine kinase

**DOI:** 10.1038/s41467-017-02637-3

**Published:** 2018-01-11

**Authors:** Tobias Pflüger, Camila F. Hernández, Philipp Lewe, Fabian Frank, Haydyn Mertens, Dmitri Svergun, Manfred W. Baumstark, Vladimir Y. Lunin, Mike S. M. Jetten, Susana L. A. Andrade

**Affiliations:** 1 Institute for Biochemistry, University of Freiburg, Albertstr. 21, Freiburg, 79104 Germany; 2 European Molecular Biology Laboratory, Hamburg Unit, EMBL c/o DESY, Notkestr. 85, Hamburg, D-22603 Germany; 3 Center for Medicine, Institute for Exercise and Occupational Medicine, Medical Center, University of Freiburg, Hugstetterstr. 55, Freiburg, 79106 Germany; 4 Faculty of Medicine, University of Freiburg, Freiburg, 79106 Germany; 5 Institute of Mathematical Problems of Biology RAS, Keldysh Institute of Applied Mathematics of Russian Academy of Sciences, Vitkevicha str. 1, Pushchino, 142290 Russia; 6 Department of Microbiology, Radboud University, Institute for Water and Wetland Research, Heyendaalseweg 135, Nijmegen, NL-6525 AJ The Netherlands; 7 BIOSS Centre for Biological Signalling Studies, University of Freiburg, Schänzlestr. 1, Freiburg, 79104 Germany

## Abstract

Sensing and uptake of external ammonium is essential for anaerobic ammonium-oxidizing (anammox) bacteria, and is typically the domain of the ubiquitous Amt/Rh ammonium transporters. Here, we report on the structure and function of an ammonium sensor/transducer from the anammox bacterium “*Candidatus* Kuenenia stuttgartiensis” that combines a membrane-integral ammonium transporter domain with a fused histidine kinase. It contains a high-affinity ammonium binding site not present in assimilatory Amt proteins. The levels of phosphorylated histidine in the kinase are coupled to the presence of ammonium, as conformational changes during signal recognition by the Amt module are transduced internally to modulate the kinase activity. The structural analysis of this ammonium sensor by X-ray crystallography and small-angle X-ray-scattering reveals a flexible, bipartite system that recruits a large uptake transporter as a sensory module and modulates its functionality to achieve a mechanistic coupling to a kinase domain in order to trigger downstream signaling events.

## Abstract (web summary)

For anammox bacteria, the sensing and uptake of ammonium is essential and specialized proteins, like *Ks*-Amt5, mediate such processes. Here, authors perform biophysical, biochemical, and structural analysis on *Ks*-Amt5 and establish a role for this protein as an ammonium-sensing signal transducer.

## Introduction

Since its discovery in 1994, the metabolic pathway of anaerobic ammonium oxidation (anammox) has received considerable attention owing to the extraordinary physiology of anammox bacteria that belong to the planctomycetes clade. In the absence of molecular oxygen as a terminal electron acceptor, anammox bacteria produce metabolic energy by coupling the reduction of nitrite (NO_2_^–^) via nitric oxide (NO) to the oxidation of ammonium (NH_4_^+^). The compounds are reacted to hydrazine (N_2_H_4_), and subsequently oxidized to the stable end product dinitrogen (N_2_)^1^. The anammox pathway has been recognized as a major contributor to clearing reactive nitrogen species from the environment, leading to the development of large-scale anammox bioreactors to remedy nitrogen pollution from the excessive use of nitrogen fertilizers in industrial agriculture. Consequently, ammonium is a major nutrient for anammox organisms, and with a catabolism directly dependent on the ammonium level inside their unique organelle, the anammoxosome, the efficient regulation of ammonium trafficking is a vital task. The metagenome of the prototypic planctomycete “*Candidatus* Kuenenia stuttgartiensis” encodes seven putative orthologs of the ubiquitous ammonium transport proteins of the Amt/Rh family^2^. Of these, *kust_*3690^3^, hereafter designated *Ks*-Amt5, uniquely features an extended, cytoplasmic domain with high homologies to histidine kinases (HK) of the HisKA subfamily^4^. Naturally occurring sequences combining Amt modules with other domains were observed earlier^5^, but no member of this class was characterized to date. In addition, no ortholog of the common interaction partner of Amt proteins, GlnK, is encoded near the *amt5* gene^6^. Sensor histidine kinases typically comprise two structurally distinct functional units; a variable N-terminal ligand-binding domain (sensor) to detect a particular signal, and a C-terminal, catalytic HK domain (transducer). Signal reception triggers conformational changes in the sensor that are transmitted to the HK domain, where the globular Catalytic and ATP-binding (CA) subdomain (PFAM 02518) binds Mg-ATP and transfers its γ-phosphate to a conserved histidine residue localized within an H-box motif in the helical Dimerization Histidine phosphotransfer (DHp) subdomain (PFAM 00512). HKs can then transfer the phosphoryl group to a conserved aspartate residue in a cognate response regulator (RR), enabling the translation of extracellular stimuli into an adequate cellular response and rapid adaptation to environmental changes^7^. During the last years, structural snapshots of a number of isolated histidine kinases^8^ or complete cytoplasmic domains^9^ have highlighted conformational changes of the proteins upon autophosphorylation or phosphotransfer events. Various structures have also been solved for isolated sensor or linker domains, such as the PAS^9–11^, GAF^12^, or HAMP domains^13^, suggesting rotations or tilts of the adjacent helices that result in variable packing and symmetry states^14^. Together with bioinformatics, genetics, and biochemical studies, such structures provide significant insight into signal reception and transduction events^15–17^. However, the molecular basis of transmembrane signal reception and transduction remains unclear. In most cases, the identity of the signal is unknown and the intrinsic flexibility of these modular proteins poses a major obstacle to solving complete, intact structures in different conformations.

Although the domain organization of *Ks-*Amt5 resembles histidine kinases of the BceS, VanS, or DesK type^18^, the protein lacks other common motifs of sensor histidine kinases that mechanistically link sensor and transducer, such as the PAS, HAMP, or STAC domains^19^. This suggested that *Ks-*Amt5 might instead have recruited and modified an existing uptake transporter module, the N-terminal ammonium transporter domain, to serve as a sensor coupled directly to the HK domain, thus defining a non-standard type of sensor-transducer system. In order to establish the role of *Ks*-Amt5 as an ammonium-sensing signal transducer based on a repurposed membrane transport protein we have produced and isolated the protein in a detergent-solubilized form and have combined a variety of techniques for the biochemical, electrophysiological, and structural characterization of *Ks*-Amt5 and its individual modules. We find that the canonical ammonium transporter module in *Ks*-Amt5, has been repurposed to selectively bind ammonium cations in a binding pocket that is not present in orthologues described previously. Electrophysiology shows that this leads to a loss of the transport function, but allows for ion binding to serve as a trigger for activating the fused histidine kinase domain.

## Results

### Kinase activity of *Ks*-Amt5 and response to NH_4_^+^

**Fig. 1:**
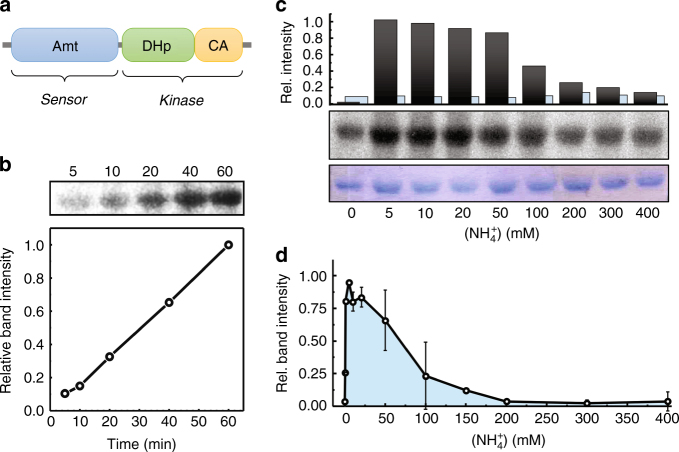
Architecture of *Ks*-Amt5 and its kinase activity. **a** The ammonium sensor/transducer *Ks*-Amt5 comprises a membrane-integral sensor domain (Amt) and a soluble, C-terminally fused histidine kinase, consisting of a DHp and a CA subdomain. **b** With [γ-^32^P]-ATP, the isolated HK domain alone shows increasing, but unregulated autophosphorylation over time. **c** In contrast, only full-length *Ks*-Amt5 reacts to variations in [NH_4_^+^] and shows rapidly increasing phosphorylation levels (black bars) at low [NH_4_^+^] that drop again at higher concentrations, whereas protein levels (Coomassie-stained, blue bars) remain constant. All data points were measured after 60 min incubation. **d** In an average of multiple (*n* = 5) experiments on a linear time scale, variations in phosphorylation levels of residue H406 show the rapid response of *Ks*-Amt5 at low [NH_4_^+^], followed by a slower decrease towards high concentrations of substrate, where passive diffusion of NH_3_ can lead to unregulated permeation across the lipid bilayer

*Ks-*Amt5 is a 679 amino-acid protein organized into an N-terminal ammonium transport (Amt) domain connected to a C-terminal domain of 269 residues (Fig. 1a, Supplementary Figure 1). This part of the protein has features of a histidine kinase, composed of a DHp and a CA subdomain with strong homologies to the cytoplasmic HK TM0853 from *Thermotoga maritima* (Supplementary Figures 1, 2)^20^. Pure protein was obtained by recombinant production in *Escherichia coli* and isolated from detergent-solubilized membranes. It eluted as a trimer in size-exclusion chromatography (Supplementary Figure 3), in line with the strictly trimeric architecture observed for all Amt family members^2^. In contrast, the large majority of known HKs function as dimers through *cis*- or *trans* autophosphorylation upon dimerization^8^. An exceptional, monomeric HK was reported recently^21^, but to our knowledge the association of HK modules in a trimeric protein core is unprecedented.

The domain arrangement of *Ks*-Amt5 suggested a coupling of ammonium transport to histidine phosphorylation that we set out to verify experimentally. In order to study the isolated HK domain, a construct producing the soluble *Ks-*Amt5^426–679^ was generated (Supplementary Table 1, Supplementary Figure 3). The protein was insensitive to Mg-GTP, but bound Mg-ATP and Mg-ATPγS with high affinities, as confirmed by isothermal titration calorimetry (*K*_D_^ATP^ = 11.16 ± 0.03 μM and *K*_D_^ATPγS^ = 36.74 ± 0.05 μM (Supplementary Figure 4)). The in vitro kinase activity of full-length *Ks-*Amt5 and its isolated HK domain were then verified using radioactively labeled [γ-^32^P]-ATP. Here, an increase of phosphorylation of the HK domain was observed over time (Fig. 1b). In contrast, a H460A variant showed residual, albeit decreased intensity of the radioactive protein bands, probably owing to unspecific phosphorylation, but a chemical stability assay under acidic and basic conditions^22^ confirmed the presence of acid-sensitive phosphoamidates (P~His) in the protein. Although ammonium ions had no observable effect on the phosphorylation levels of the HK fragment, full-length *Ks*-Amt5 exhibited a strong modulation of the kinase activity in the presence of the cation (Fig. 1c), in that phosphorylation levels showed a steep increase at low concentrations of NH_4_^+^, followed by a decrease at high concentrations (Fig. 1d). This is in line with a sensor optimized for low external ammonium levels, whereas the modulation observed at higher concentrations may not be physiologically relevant. Note, however, that sudden, high levels of the cation will also lead to an increased passive diffusion of membrane-permeable ammonia, NH_3_, which is in equilibrium with NH_4_^+^ at a pK_a_ of 9.25^23^ and will alleviate the need for active uptake. These results establish a mechanistic link between NH_4_^+^ sensing in the Amt domain and modulation of P~H406 in the HK transducer domain, confirming *Ks*-Amt5 as a unique type of sensor-transducer.

### *Ks*-Amt5 binds but does not translocate NH_4_^+^ ions

**Fig. 2:**
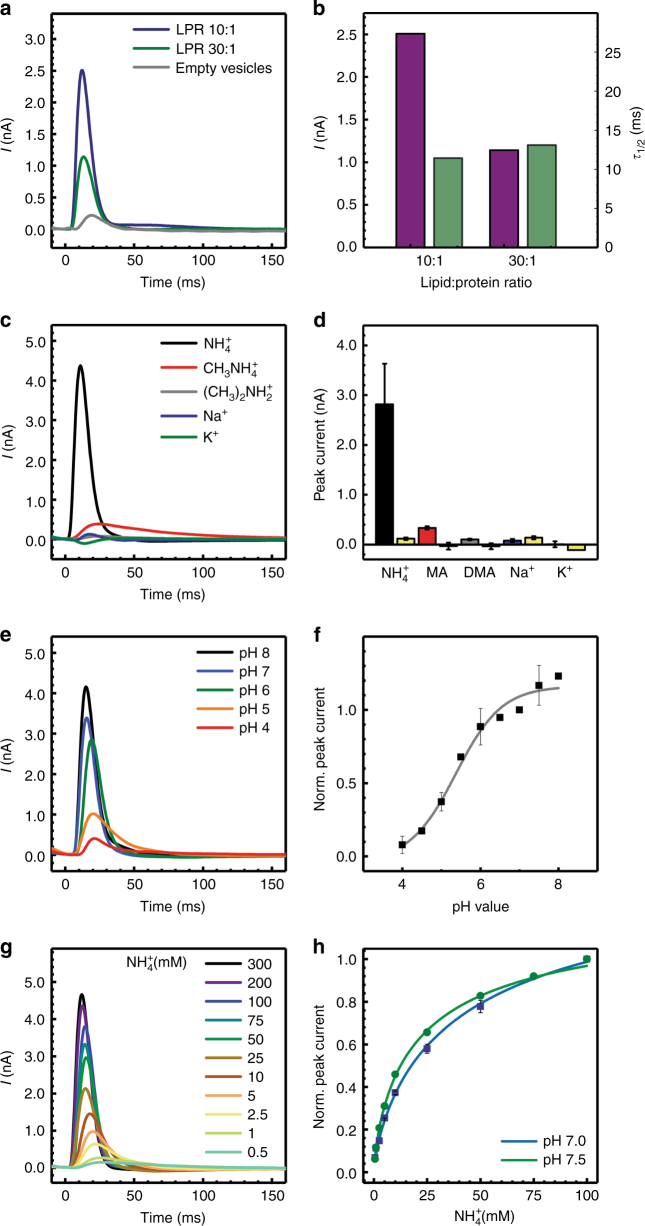
Electrophysiological analysis of *Ks*-Amt5 in proteoliposomes. **a** Transient currents recorded at lipid:protein ratios (LPR) of 10:1 and 30:1 vs. protein-free liposomes reveal specific charge displacement events in the presence of *Ks*-Amt5. **b** Bar graph of the peak current (violet, nA) and half-maximum decay time (green, ms) values from **a**. The unchanged decay time points towards charge displacement rather than ion translocation. **c** Transient currents recorded at pH 7.5 for different cations (300 mM) at a LPR of 10:1. *Ks*-Amt5 is highly specific for NH_4_^+^, but does not act on methylammonium (MA) or dimethylammonium (DMA), which are alternative substrates of assimilatory Amt proteins. **d** Bar graph of the peak currents measured in **c** and the respective backgrounds (yellow). Only NH_4_^+^, but not MA or DMA, triggered an electrogenic response. **e** pH-dependent transients induced by 300 mM NH_4_^+^ concentration jumps. **f** Variation of normalized peak currents with pH (as in **e**). The fit (solid line) used a titration function^70^ yielding a pK_app_ of 5.5. **g** Saturation behavior of NH_4_^+^ transients at pH 7.5 with increasing concentration of the ion. **h** Normalized peak currents with respect to NH_4_^+^ concentration at pH 7.5 and 7.0. The affinity for the cations is increased at the higher pH value (*K*_0.5_^app^, pH 7.5 = 22.9 ± 0.87 mM fitted with a Hill coefficient of 0.75). All experiments were done at least in triplicate on two different protein reconstitutions and two separate protein batches. Error bars are thus calculated from a minimum of *n* = 12

We proceeded to reconstitute detergent-solubilized *Ks-*Amt5 and its isolated Amt domain, *Ks*-Amt5^1–408^ into proteoliposomes (Supplementary Table 1, Supplementary Figure 3), for direct analysis of ion transport by solid-supported membrane (SSM)-based electrophysiology. Both constructs showed identical, NH_4_^+^-dependent transient current responses (Fig. 2a, Supplementary Figure 5a, b), as well as an unprecedented absence of a response to methylammonium (MA, H_3_C–NH_3_^+^) that is a typical alternative substrate of Amt proteins (Fig. 2c). With assimilatory Amt proteins from *Archaeoglobus fulgidus*, MA transport rates of 50–85 % of those recorded for ammonium were obtained^24^. In the case of *Ks*-Amt5, however, MA, dimethylammonium, Na^+^ and K^+^ produced only background traces (Fig. 2c, d). Note that in numerous earlier studies the uptake of radioactive ^14^CH_3_–NH_3_^+^ was used to characterize Amt/Rh transport, but in light of the innate selectivity seen here, functional interpretations derived from such assays may not be valid for all members of the Amt/Rh family. The current responses of *Ks*-Amt5 also differed characteristically from those recorded for the assimilatory proteins from *A*. *fulgidus*^24^. Although the peak currents decreased from lipid:protein ratios (LPR) of 10:1–30:1, the half-maximum decay time τ_½_^25^ remained unchanged for both the Amt domain (*Ks*-Amt5^1–408^) and the full-length construct (Fig. 2a, b; Supplementary Figure 5a, b). Similar observations were made earlier for lactose permease LacY^26^ and melibiose permease MelB^27^ and were attributed to a complex electrogenic process comprising a minor, transport-related and a major, charge-induced conformational rearrangement. In contrast, the translocation of an ionic species is characterized by extended τ_½_ times at higher LPR, as previously reported for the assimilatory Amt1 from *A*. *fulgidus* (Supplementary Figure 5c, d)^24^. Accordingly, the transient currents recorded on *Ks-*Amt5 in the presence of NH_4_Cl at various pH values (Fig. 2) arise predominantly from charge displacement events: The Amt domain shows high selectivity for NH_4_^+^ and binds the ion specifically, but it does not efficiently mediate its translocation across the membrane. With increasing NH_4_Cl concentrations, this process showed saturation behavior at pH of 7.0 and 7.5 (Fig. 2g, h), with an apparent half-saturating concentration of the protein for the cation of *K*^app^_0.5_ = 22.9 ± 0.9 mM (pH 7.5) and a pK_app_ = 5.5 (Fig. 2e, f). In order to further characterize the binding of NH_4_^+^ to the Amt module, we proceeded to carry out biophysical binding studies. As titration calorimetry did not yield useful results for the membrane domain, we instead conducted microscale thermophoresis (MST) studies that revealed a binding event for NH_4_^+^ with an apparent *K*_*d*_ = 41 ± 24 µM, whereas MA only bound with *K*_*d*_ = 2350 ± 440 µM (Supplementary Figure 6). This is well in line with the results from electrophysiology, as well as with a strong preference for ammonium over MA.

### Three dimensional structure of *Ks*-Amt5

**Fig. 3:**
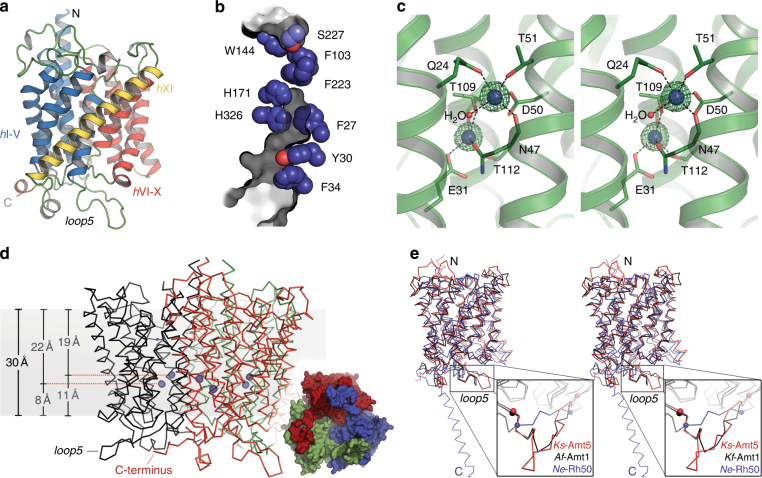
High-resolution structure of the Amt domain of *Ks*-Amt5. **a** The monomer of *Ks*-Amt5 exhibits the typical transporter fold of Amt/Rh family proteins, with two groups of five transmembrane helices (*h*I-V, *h*VI-X) related through a twofold pseudosymmetry axis in the membrane plane, stabilized through the clamping helix *h*XI. The C-terminal HK domain was undefined due to structural disorder. **b** The Amt domain retains all functionally relevant residues (orientation as in **a**), in particular the selectivity filter (W144/S227), the Phe gate (F103, F223) and the His pair (H171, H326). However, in comparison with the *Af*-Amt1 transporter, for which the transport channel is overlaid in gray, an inward shift of the His pair in combination with three bulkier residues (F27, Y30, F34) in helix *h*I effectively closes the transmembrane transport pathway for ammonium ions. **c** Stereo representation of the NH_4_^+^ binding sites within the membrane, with an *F*_o_–*F*_c_ omit difference electron density map contoured at the 5.0 σ level. See Supplementary Figure 7 for the relative orientation of the ion binding site and the transport channel. **d** Side view of the trimer, indicating the location of the NH_4_^+^ binding sites within the membrane (gray). The inset shows a bottom view of the trimer, highlighting how the C-termini extend over the neighboring monomer towards loop 5. **e** Superposition of *Ks*-Amt5 (red) with *Af*-Amt1 (black) and the Rh orthologue *Ne*-Rh50 (blue). The latter contains a helical extension at the C-terminus that adopts a different conformation than the connection of the HK domain in *Ks*-Amt5. Although the three structures are homologous in the membrane-integral part, they differ strongly in length and conformation of the functionally relevant loop 5 (bordered by balls) that connects the symmetry-related halves of each monomer

As ammonium did not affect the HK domain directly, the activation of the transducer must involve a conformational change of the Amt receptor upon signal recognition. For bacterial assimilatory Amt proteins, a long-standing debate concerning their functionality as electrogenic transporters for NH_4_^+^ was settled only recently^24^, and we furthermore provided evidence for structural changes during transport by constructing an Amt-GFP chimera from plant and yeast, the engineered ammonium sensor AmTrac^28^, so that ammonium-triggered structural changes in Amt proteins are a reasonable assumption. For the bipartite *Ks*-Amt5 system, the conformational coupling of the two domains requires intrinsic structural flexibility that seems to be a hallmark of sensor/transducer systems. In spite of this potential obstacle we successfully generated well-diffracting single crystals of the full-length protein. *Ks*-Amt5 crystallized in space group *P*6_3_ and crystals diffracted to a resolution better than 2 Å using synchrotron radiation. The structure was solved and refined to 1.98 Å resolution, but only the transmembrane Amt domain was well defined in the electron density maps. The detailed atomic model covered amino-acid residues 1–405 up to the beginning of the helical connector between the two domains (Supplementary Table 2). From here on, the quality of the electron density map deteriorated due to structural disorder of the HK domain, and although various features such as extended helices were discernible, an atomic model could not be built. The flexibility of the kinase domains is a common feature of signaling proteins, so that this level of disorder was not unexpected. In many analyses of sensor histidine kinases, however, the transmembrane segments are either truncated or are small per se, so that the kinase forms the crystal lattice. In our case, it was the exceptionally large membrane-integral Amt domain that formed crystal contacts, allowing instead for an excellent insight into signal recognition by the sensor. The transmembrane domain of *Ks*-Amt5 follows the Amt/Rh fold (Fig. 3a), aligning to *A*. *fulgidus* Amt1 with a root-mean-squared deviation of all-atom positions of 1.2 Å (Fig. 3e). Although ammonium ions are not efficiently translocated by *Ks*-Amt5, the known structural features of Amt transporters are fully conserved. They include a pseudo-twofold symmetry axis relating helices I-V and VI-X around the central translocation pore, connected by the extended loop 5 in the cytoplasm, as well as a long helix XI that stabilizes the two halves (Fig. 3a). As expected from primary structure alignments (Supplementary Figure 1), all known functional motifs are retained, including the selectivity filter (W144, S227), the Phe gate (F103, F223) and the His pair (H171, H326) (Fig. 3b)^29^. However, the model shows the translocation pathway obstructed not only by the Phe gate as found in assimilatory Amt/Rh proteins^23, 30, 31^, but also by the bulkier side chains of residues F27, Y30, and F34 that in *Ks*-Amt5 occupy the respective positions of residues V23, F26, and M30 in *Af*-Amt1, and by a shift of the His pair of ~ 0.8 Å into the channel (Fig. 3b). This arrangement leaves the selectivity filter vestibule (W144 and S227) intact, but likely prevents NH_4_^+^ from reaching the other side of the membrane. Residue H326 is linked via H-bonding across two putative water molecules to an unprecedented cation-binding site. It is most uncommonly situated within the membrane and may represent the selective high-affinity NH_4_^+^-binding site indicated by our functional studies (Fig. 3c, Supplementary Figure 7). Here, two ammonium cations are tightly coordinated by a network of residues, namely Q24 and E31 from helix *h*1, N47, D50 and T51 from *h*2 and T109 and T112 from *h*4 (Fig. 3c). The binding site is located ~ 10 Å from the membrane boundary (Fig. 3d), and only two amino acids in the wider proximity (E31 and D50) are acidic, providing charge compensation for two monovalent cations. The observed binding geometry with five ligands on each cation is distinct from the octahedral symmetry commonly observed for Na^+^, and the short bond distances of 2.2–2.4 Å are in excellent agreement with calculated and observed coordination distances of water to NH_4_^+^, making the assignment unambiguous^32^. *Ks*-Amt5 thus diverts the substrate cations that have passed the selectivity filter (W144, S227) into high-affinity binding sites, where binding may lead to structural rearrangements that trigger signaling. Through the charged residues, E31 and D50 and their unusual location within the membrane (Fig. 3c, d) the binding sites select for charged species, rationalizing the pH-dependence of the current response (Fig. 2e, f), as well as the observed incomplete translocation of charge (Fig. 2a). The structural data now allow for investigating whether the rearrangement of charged residues in this binding site could be the origin of the charge displacement observed in the transient current profiles and eventually result in kinase activation.

### Structure of the full-length signaling protein

**Fig. 4:**
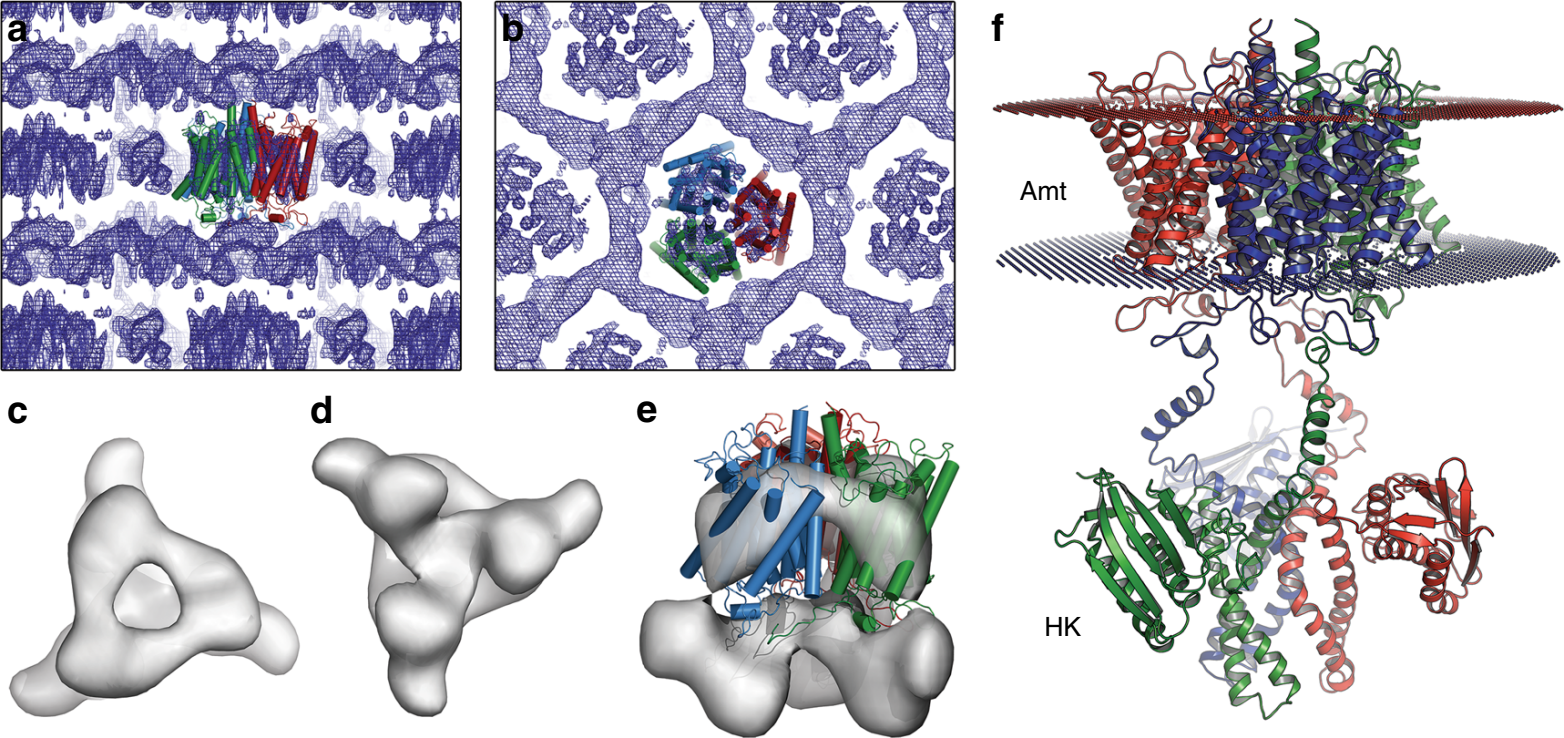
Low-resolution analysis of full-length *Ks*-Amt5. **a** Electron density map showing the strata of the type I crystals, with the high-resolution structure of the Amt domain placed and shown in cartoon representation. **b** Top view of **a**. The electron density map is better resolved for the Amt domain that determines the crystal packing, whereas the HK domain shows disorder and attains a position between neighboring Amt domains. **c** Top view of full-length *Ks*-Amt5 in a weighted electron density synthesis at 8 Å resolution contoured at the 1σ level. **d** Bottom view onto the HK domain. **e** Low-resolution electron density synthesis of *Ks*-Amt5, superimposed with the high-resolution 3D structure of the Amt domain. **f** Hypothetical, extended arrangement of the sensor-transducer in the membrane, showing the structure of the Amt domain linked to a homology model for the HK domain in an arbitrary, extended conformation. The HK domains are structurally flexible in the cytoplasm, and their conformation in solution is not identical to the one observed in the crystal lattice (Supplementary Figure 10a)

**Fig. 5:**
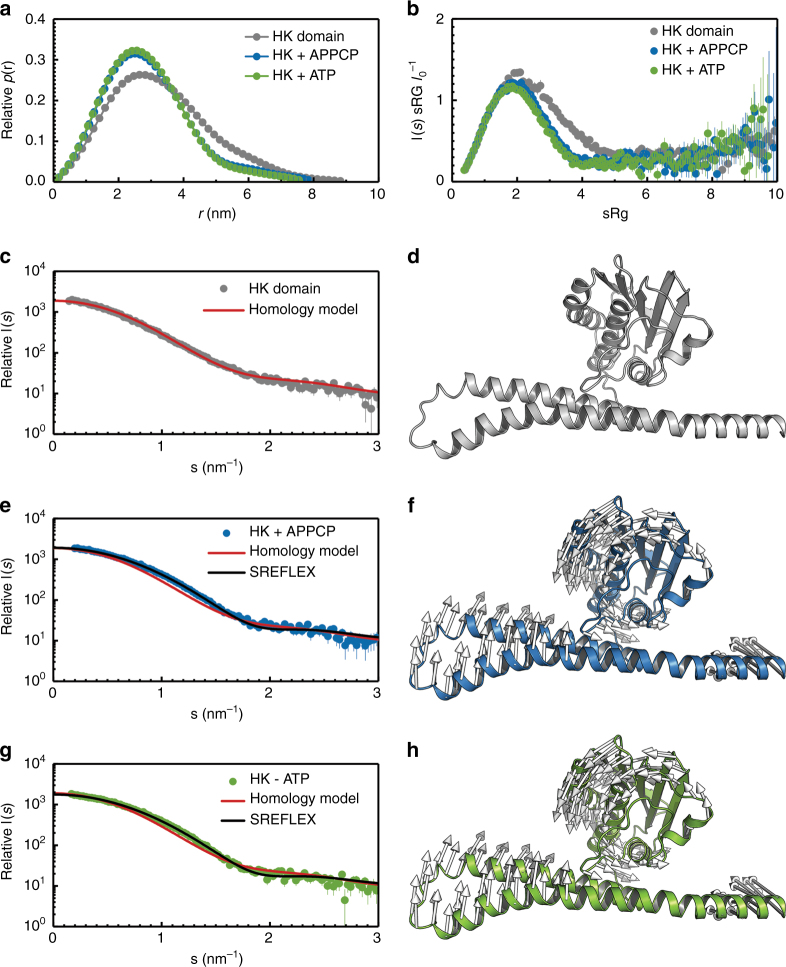
SAXS analysis of nucleotide binding to the HK domain. Real-space distance distributions *p*(*r*) of the HK domain with no ligand (gray) and in the presence of 10 µM of APPCP (blue), or 10 µM of ATP (green). **b** Dimensionless Kratky plot of the data in **a**, highlighting the change toward a more compact and less-flexible structure when either APPCP or ATP are added to the HK domain. **c** SAXS data for the ligand-free HK domain, fitted with the homology model depicted in **d**. **e** SAXS data for the Mg-APPCP-bound HK domain, fitted with the homology model (red), as well as with the best SAXS-refined elastic network conformation (black). **f** Conformational transitions from the template homology model refined against the Mg-APPCP-bound data in **e**. Vectors describe the direction of motion. **g** SAXS data and corresponding fits as in **e** for the Mg-ATP bound HK domain. **h** The derived conformational transitions from **g** are virtually identical to the ones obtained for Mg-APPCP in **f**. No effect was noted upon addition of the protein kinase C inhibitor Ro 31-8220

In Amt/Rh proteins, loop 5 forms the connection of the two symmetry-related halves of the integral membrane protein and was suggested to convey flexibility for an intrinsic movement during transport^23, 33^. This was supported by the generation of the ammonium sensor AmTrac, where a circularly permutated fluorescent protein was inserted in this position^28^. Contrary to other known structures of Amt/Rh family members, where loop 5 commonly encompasses 10–12 amino acids, *Ks*-Amt5 contains a substantially longer stretch of 19 residues, G186–N205 (Fig. 3e). In the trimer, this elongated loop 5 region is in close proximity to the extended C-terminus of the neighboring monomer and thus the HK module, representing a possible mechanistic coupling between the two domains (Fig. 3d). However, as crystal packing interactions presumably nudged the HK domains into a non-physiological position within the crystal lattice (see below), the potential role of loop 5 and of ammonium binding to the cation-binding site in signal transduction between the domains will require further study. SDS-PAGE analysis of washed crystals dissolved in buffer confirmed the presence of the entire polypeptide chain, but extensive attempts to lock the HK domain into a defined position via co-crystallization or soaking using Mg-ATP, ADP, or non-hydrolysable ATP analogs did not yield a defined conformation for the entire sensor/transducer assembly. However, the flexibility of the HK domain in the crystals decreased slightly when the protein kinase C inhibitor Ro 31–8220^34^ was used as a crystallization additive, revealing additional electron density features for the cytoplasmic domain (Fig. 4a, b). Complete X-ray data sets with the compound were collected to an extended lower resolution limit of 600 Å to allow for ab initio phasing^35^, yielding an electron density envelope of the full-length protein at 8.0 Å resolution (Fig. 4c, d, Supplementary Table 3). Here, the two domains of *Ks*-Amt5 were clearly defined, and the HK monomers were positioned apart (Fig. 4). Crystal lattice contacts consisted exclusively of direct interactions of the Amt domains, placing the HK domains in an environment constrained by the detergent micelles of neighboring Amt domains, further adding to the intrinsic flexibility of these proteins and the observed partial disorder in the structure. In an attempt to overcome this influence of crystal packing on the protein conformation and obtain low-resolution structures of *Ks*-Amt5 in solution that could help us rationalize the effect of ligands, we proceeded to use small-angle X-ray scattering (SAXS), for which the detergent was changed to CYMAL-5 to minimize background scattering (see Supplementary Methods, Supplementary Figure 8). The molecular weight of all particles estimated from the SAXS data agreed well with data from size-exclusion chromatography for a trimeric *Ks*-Amt5 solubilized in detergent (Supplementary Figure 9, Supplementary Table 4). In contrast, the isolated *Ks*-Amt5^426–679^ fragment of the HK domain behaved as a monomer in solution (Fig. 5, Supplementary Table 5). Interestingly, in the absence of nucleotides, a homology model based on the high-resolution crystal structure of *Tm*-HK0853^20^ adequately described the *Ks*-Amt5^426–679^ solution data, whereas in the presence of Mg-ATP or non-hydrolysable Mg-APPCP this model represented a poor fit. Normal mode calculations and automated domain partitioning and exploration of conformations guided by the SAXS data revealed a more compact protein model (Fig. 5, Supplementary Table 5). This indicates that the nucleotide binding events detected by ITC (Supplementary Figure 4) can induce conformational alterations in the HK module. For the full-length protein, however, best fits were obtained for oblate structures, in which the HK domains are positioned directly below the plane of the membrane, facing apart. Based on the conformational plasticity of the system, an ensemble optimization was conducted on the data, revealing a bimodal distribution of the gyration radii, highlighting the flexible nature of the protein (Supplementary Figure 10). Addition of nucleotides and/or ammonium at this stage led to protein aggregation, but nevertheless the data indicate that—owing to the detergent micelle—solubilized *Ks*-Amt5 attains a similar, compact conformation in solution as it does in the crystal, whereas a more extended conformation is expected in vivo, such as the one represented by a molecular model created from the Amt domain structure and a homology model of the kinase domain (Fig. 4f). In view of recent structural and biochemical data linking the mode of autophosphorylation (*cis* or *trans*) to the handedness of the two helices in the DHp subdomain and the length of their linker loop^14, 36^, we hypothesize that in its natural membrane environment, the transducer domain of *Ks*-Amt5 may transiently form dimers in a concerted way and carry out a *cis* autophosphorylation reaction.

## Discussion

*Ks*-Amt5 represents the prototype of a modular sensor-transducer system that evolved from the widespread ammonium transporter role to serve as a specific receptor for the NH_4_^+^ cation. For activation, *Ks*-Amt5 initiates a transport cycle at the selectivity filter, but then diverts two substrate cations into a high-affinity binding site within its membrane core that is absent in assimilatory Amt proteins. Considering that ammonium ions represent a bulk nutrient in the anammox pathway of “*Ca*. K. stuttgartiensis”, the presence of a two-ion ammonium binding site in the Amt domain may be part of a mechanism of signal integration that leads to stable kinase activation only at relevant concentrations of the ion (Fig. 1c). Lacking a typical transducer module such as a HAMP domain, structural rearrangements that are likely caused by populating this binding site must suffice to activate the fused HK domain. Our structural data suggest that the HK domain of one monomer is positioned below the neighboring monomer within the functional trimer, which necessarily brings the C-terminal linker region between both domains into close contact with the adjacent loop 5 (Fig. 3d), and it is plausible to assume that rearrangements of loop 5 may be communicated to the kinase. Earlier studies on plant and bacterial Amt proteins have revealed the importance of an interaction between the loop 5 region and the C-termini of neighboring monomers for Amt transport, resulting in an allosterically regulated transport behavior^33, 37^. *Ks*-Amt5 thus re-purposes a nutrient uptake transporter by creating an original, possibly cooperative substrate recognition site that triggers the transduction of conformational information to a fused transducer and, in turn, to a yet to be identified RR in the cytoplasm (karyoplasm) of the planctomycete *K*. *stuttgartiensis*.

## Methods

### Cloning and heterologous gene expression

The 2037 bp *Ks*-*amt5* gene was amplified from genomic DNA of “*Candidatus* Kuenenia stuttgartiensis” by polymerase chain reaction, using primers F1 and R1 (Supplementary Table 1), and ligated into the pCR2.1-TOPO plasmid (Thermo Fischer) via the BamHI and KpnI restriction sites. For protein production, the *amt5* gene was re-cloned by PCR using primers F2 and R2 (Supplementary Table 1) and ligated into pET21a (Novagen) using *Nde*I and *Xho*I restriction sites. The resulting plasmid, pET21a::Ks-*amt5*, expressed full-length Amt5 with a short amino-acid linker (LE) followed by a C-terminal His_6_-tag. To isolate the Amt membrane domain, a new construct (comprising residues M1 to A408) was produced by Gibson assembly, using primers F2 and R3 (Supplementary Table 1) and pET21a::Ks-*amt5* as template^38^. The resulting pET21a plasmid produced an Amt domain without any linker before the C-terminal His_6_-tag. The cytosolic domain fragment (comprising residues L426 to K679) was cloned into pET15 (Novagen) modified to carry a His_10_-tag. pET21a::Ks-*amt5* was again used as template for a PCR reaction with primers F3 and R4 carrying NdeI and SacI restriction sites (Supplementary Table 1). The final construct, pET15dt::Ks-*amt5*^426–679^, encoded for an N-terminal His_10_-tag followed by a Tobacco Etch Virus (TEV) protease recognition motif (ENLYFQG) and a NdeI restriction site insertion, such that upon TEV cleavage the isolated HK module retained three additional residues (GHM) before residue L426.

Protein variants were produced by site-directed mutagenesis using Pfu-Turbo polymerase (Stratagene) and the respective primers and template vectors (Supplementary Table 1) by PCR. DpnI (Fermentas) was used to digest the methylated DNA template. All constructs were verified by DNA sequence analysis (GATC Biotech).

*E*. *coli* C43(DE3) (Lucigen) was used to produce the full-length protein, the Amt domain plus respective variants, while *E*. *coli* BL21(DE3) (New England Biolabs) was used for the production of the soluble domain *Ks*-Amt5^426–679^ and variants thereof. Cells were cultured in LB medium at 37 °C, supplemented with 100 µg mL^–1^ ampicillin, in baffled Erlenmeyer flasks under agitation at 180 rpm. Gene expression was induced at an OD_600_ of 0.4–0.6 by addition of 0.4 mM isopropyl-β-*D*-thiogalactopyranoside. When producing full-length protein, the culture temperature was lowered on ice for 10 mins before induction and cultivation was then continued for 16 h at 20 °C. To induce the production of *Ks*-Amt5^426–679^, the culture temperature was lowered to 30 °C for 3–4 h after induction. Cells were harvested by centrifugation at 4 °C, frozen in liquid nitrogen and kept at –80 °C until further use.

### Protein purification

Cell pellets were thawed, resuspended in buffer A (20 mM Tris/HCl pH 8.0, 500 mM NaCl and 40 mM imidazole) and disrupted using a Microfluidizer M110P (Microfluidics). To isolate the membrane fractions containing *Ks*-Amt5 or *Ks*-Amt5^1–408^, the broken cells were first sedimented at 30,000 × *g* for 40 min and the resulting supernatant was subjected to an ultracentrifugation at 300,000 × *g* for 1 h. The membrane fraction was resuspended in buffer A supplemented with 10 % (*v/v*) glycerol and proteins were solubilized with 1 % (*w/v*) dodecyl-β-*D*-maltopyranoside (DDM, Anatrace). The insoluble material was removed by centrifugation at 108,000 × *g* for 40 min and the remaining crude membrane protein extract was loaded onto a Ni-affinity column (His Trap HP, GE Healthcare), equilibrated with buffer A supplemented with 10 % (*v/v*) glycerol and 0.03 % (*w/v*) DDM. Detergent exchanges were carried out during affinity chromatography. Protein impurities were subsequently removed by step increments in buffer B (20 mM Tris/HCl pH 8.0, 500 mM NaCl and 500 mM imidazole pH 8.0) supplemented with 10 % (*v/v*) glycerol and the chosen detergent. *Ks*-Amt5 and the Amt domain were selectively eluted with 50 % buffer B. The protein-containing fractions were concentrated by ultrafiltration (Sartorius Vivaspin) with a molecular weight cut-off (MWCO) of 100 kDa (for the full-length protein) or 50 kDa (for the Amt domain), and subsequently loaded onto a Superdex 200, 26/60 size-exclusion column (GE Healthcare), equilibrated in buffer C (20 mM HEPES/NaOH pH 7.5 or 20 mM Tris/HCl pH 8.0 with 100 mM NaCl), supplemented with 5–10 % (*v/v*) glycerol and the chosen detergent. Fractions containing trimeric *Ks*-Amt5 or the Amt construct were pooled and concentrated as described above.

For isolation of the soluble *Ks*-Amt5^426–679^ domain and variants, broken cells obtained as described above were centrifuged at 108,000 × *g* for 1 h. The crude protein extract was loaded onto a Ni-affinity column (His Trap FF, GE Healthcare) equilibrated in buffer A. Impurities were removed as described above and the target protein was eluted with 50 % buffer B. Fractions containing protein were subjected to cycles of concentration/dilution in Vivaspin concentrators (Sartorius) with MWCO of 10 kDa to decrease the imidazole content in the buffer. The affinity tag was cleaved overnight at 4 °C, in the presence of 2 mM 2-mercaptoethanol and 1 mg of TEV protease per mL of protein solution. The digestion product was once more loaded onto a His Trap FF column prepared as described above and the untagged *Ks*-Amt5^426–679^ was collected in buffer A. Digested protein was concentrated and applied onto a Superdex 75 26/60 size-exclusion column (GE Healthcare) equilibrated in buffer C. The eluted, monomeric *Ks*-Amt5^426–679^ protein was pooled and concentrated as described above. All purification steps and protein handling were carried out at 4 °C. Protein purity was monitored by SDS-PAGE and protein was quantified using the bicinchoninic acid protein assay. If not used immediately, aliquots of concentrated protein were frozen in liquid nitrogen and stored at –80 °C.

### Isothermal titration calorimetry

Calorimetric titrations were carried out at 20 °C with a VP-ITC microcalorimeter (MicroCal/GE Healthcare), following established protocols^6, 39^. The sample cell contained 100 µM of *Ks*-Amt5^426–679^ in 1.437 mL of buffer solution consisting of 50 mM Tris/HCl pH 8.0, 100 mM NaCl and 2 mM MgCl. Solutions of 1.3 mM of potential ligands such as ATP, ADP, GTP (Jena Biosciences) or ATP-γS (AppliChem) were freshly prepared in the same buffer. All solutions were degassed and incubated at 20 °C before the titration runs. After the first ligand injection of 4 µL, 20 injections of 14 µL each or 28 injections of 10 µL each were titrated into the sample cell while stirring at 300 rpm. The total time between two injections was 240 s. Each experiment was done in triplicates and blank assays were performed in the absence of protein under otherwise identical experimental conditions. The titration peaks were converted to heat and plotted as a function of the molar ratio of ligand to protein after volume displacement corrections. Data points were integrated and fitted in NITPIC^40^ and SEDPHAT^41^ using a single-binding-site model. Final figures were prepared using GUSSI^42^.

### Microscale thermophoresis

*Ks*-Amt5 was labeled using the Monolith Protein Labeling Kit RED-tris-NTA (NanoTemper Technologies). The labeling reaction was performed according to the manufacturer’s instructions in size-exclusion buffer using a concentration of 0.2–0.5 µM protein and 0.1–0.2 µM of dye at room temperature for 30 min. Concentration series of ligands (NH_4_^+^, CH_3_NH_3_^+^, and Na^+^) were prepared using size-exclusion buffer, producing ligand concentrations ranging from 1.5 µM to 50 mM. For the measurement, each ligand dilution was mixed with one volume of labeled *Ks*-Amt5 protein. After 30 min incubation, the samples were loaded into Monolith NT.115 Premium Treated Capillaries (NanoTemper Technologies) and microscale thermophoresis (MST) was measured using a Monolith NT.115 instrument (NanoTemper Technologies) at a temperature of 22 °C. Instrument parameters were adjusted to 40 % LED power and 60 % MST power. Data of three independent measurements were analyzed using the signal from MST-off time of 2–3 s and a MST-on time of 0.5–3 s (MO.Affinity Analysis software version 2.2.7, NanoTemper Technologies). Fitting of the data was carried out in Origin 2017 (OriginLab).

### In vitro autophosphorylation activity measurements

In total, 10–15 μM of *Ks*-Amt5 or *Ks*-Amt5^426–679^ in buffer C were incubated at room temperature with a mixture containing 0.175 μCi μL^–1^ of [γ-^32^P]-ATP (3000 Ci·mmol^–1^; Perkin-Elmer) and 1 mM freshly prepared ATP in buffer D (50 mM HEPES/NaOH or Tris/HCl buffer at pH 8.0 and 100 mM NaCl, supplemented with 10 mM MgCl_2_ and 10 mM MnCl_2_). At given time intervals, 15 μL samples were removed and the reaction was stopped by addition of 5 μL of SDS-PAGE loading dye (125 mM Tris/HCl pH 6.8, 20 % (*v/v*) glycerol, 5 % SDS, 0.2 % bromophenol blue and 1 % (*v/v*) 2-mercaptoethanol), supplemented with 40 mM EDTA. The mixtures were briefly spun and 15 μL were loaded on a 10 or 12.5 % SDS-PAGE that ran for 80 min at 30 mA. The resulting gels were dried for 2 h at 80 °C and radioactive protein bands were visualized by autoradiography on an imager (FLA-3000, FujiFilm) for 12–16 h. To verify the influence of NH_4_^+^ on the phosphorylation levels of *Ks*-Amt5, 2 μL of concentrated NH_4_Cl stock solutions, prepared in buffer D, were added to vials containing 20 μL of reaction mixture. As a control, equivalent NaCl stock solutions were used instead of NH_4_Cl. The chemical stability of the phosphohistidine was monitored by heating the samples for 45 min at 37 °C in the presence of variable concentrations of NaOH or HCl solutions^22^. The resulting band intensities were imaged and quantified using ImageJ-1.48^43^. The original blots and SDS-PAGE gels are shown in Supplementary Figure 12.

### Reconstitution of *Ks*-Amt5 in proteoliposomes

Liposomes were prepared from a mixture of 25 mg mL^–1^ *E*. *coli* polar lipid extract in chloroform (Avanti Polar Lipids) and dried at room temperature under a nitrogen atmosphere in a VV Micro rotary evaporator (Heidolph). The dried lipid film was resuspended in 10 mg mL^–1^ of buffer E (100 mM K_2_HPO_4_/KH_2_PO_4_ at pH 7.5), for 60 min under rotation. The resulting suspension was extruded 13 times through a membrane with 400 nm pore size (Whatman) using a mini-extruder (Avanti Polar Lipids) and frozen in liquid nitrogen for storage at –80 °C if not used directly. For proteoliposome preparation, Triton X-100 (Anatrace) was added to the liposome suspension to a final concentration of 2 % (*v/v*) and the mixture was incubated at room temperature for 1 h. Pure protein was added to the detergent-destabilized liposomes at the desired LPR (*w/w*) and incubated for another 30 min. Detergent was subsequently removed using 13 mg of activated Bio-beads (BioRad) per 2.14 mg of Triton X-100, in three steps; two times 1 h at room temperature and overnight at 4 °C^44^. The proteoliposome suspension was collected by ultracentrifugation at 300,000 × *g* for 1 h at 4 °C, gently resuspended in buffer E to a final concentration of 10 mg mL^–1^ and either used directly or frozen in liquid nitrogen and stored in aliquots at –80 °C.

### Orientation of *Ks*-Amt5 in proteoliposomes

To investigate the orientation of *Ks*-Amt5 reconstituted in proteoliposomes, we used an assay based on the accessibility of cysteine residues to the fluorescent dye tetramethyl-rhodamine-5-maleimide (TMR, Thermo Fischer). TMR has excitation and emission maxima within the UV range and binds covalently to free cysteine residues^45^. Native *Ks*-Amt5 contains four cysteine residues, C18 (*h*I), C336 (*h*X), and C381 (*h*XI) in the Amt module and C528 in the CA domain of the HK module (Supplementary Figure 1). The variant *Ks*-Amt5 C381A/C528A was produced (Supplementary Table 1) to evaluate the accessibility of TMR to the remaining cysteine residues in the Amt membrane module. To avoid fluorescence quenching by light, samples were kept covered with aluminum foil during the assays. Typically, 10–15 μg total protein in 15 μL were incubated with 3 μM TMR for 20 min on ice. The reaction was stopped with 10 mM of dithiothreitol (DTT) and further incubated on ice for 5 min, after which SDS-PAGE loading dye was added. After another 10 min at room temperature, the mixture was spun down and 20 μL were loaded onto a 10 % SDS-PAGE mini gel. Electrophoresis was run at 25 mA for 90 min. Gels were imaged under UV light (GelDocXR, BioRad) and further stained with Coomassie brilliant blue (Supplementary Figure 11). The original blots and SDS-PAGE gels are shown in Supplementary Figure 13.

### SSM-based electrophysiology of *Ks*-Amt5 and *Ks*-Amt5^1–408^

Electrophysiology assays followed established protocols^24, 25^. In brief, 30 μL of ice-cold proteoliposome suspension containing circa 1 mg mL^–1^ of protein (LPR 10:1) were added to the reaction chamber of an SSM flow-through cuvette and allowed to adsorb for 1 h to a phosphatidylcholine:octadecanethiol hybrid bilayer covering the gold electrode surface. Experiments were performed under a constant pressure of 60 mbar at 21 °C. To record transient currents, the reaction chamber was sequentially flushed with non-active, active and non-active solution for 2.5, 2 and 2.5 s periods, respectively. The non-active solution consisted of 100 mM buffer, 500 mM KCl and 300 mM NaCl. Buffer solutions used were K_3_citrate/citric acid at pH 4.0–5.5, K_2_HPO_4_/KH_2_PO_4_ buffer at pH 6.0–7.5 or Tris/HCl pH 8.0–8.5. The active solution was prepared in 100 mM buffer, 500 mM KCl, *x* mM substrate and (300—*x*) mM of NaCl. Experiments varying pH were conducted on the same sensor after incubating each new non-active solution for 30 min^46^. Four to ten measurements were carried out in series to obtain a stable and average transient current profile. A minimum of two purification batches, two reconstitutions per batch and two sensors per proteoliposome preparation were used for statistical significance. A current amplifier (Keithley 428) was used with the gain set to 109 V/A, using a low-pass filter in the 100 Hz range.

### Protein crystallization

Full-length *Ks*-Amt5 obtained after size-exclusion chromatography was immediately used for crystallization trials at 4 °C and 20 °C, using the vapor diffusion method and a sitting drop of 2 μL, containing a 1:1 mixture of 8 mg mL^–1^ protein and variable reservoir solutions. Different detergents and detergent mixtures in the protein buffer were tested and the best results were obtained when the protein was purified in buffer containing 0.65 % (*w/v*) of nonyl-β-*D*-maltopyranoside and 0.03 % (*w/v*) DDM. Rhombohedral crystals appeared at 20 °C within fifteen days, in crystallization drops equilibrated against 300 μL of 0.1 M MES/NaOH pH 7.0 with 24 % (*w/v*) of polyethylene glycol 3350. Cryo-protective conditions were achieved through a slight increase in the drop reservoir concentration after opening the crystallization well for 5–10 min. Crystals were harvested into nylon loops (Hampton Research) directly from the drop and flash-cooled in liquid nitrogen. Attempts to trap the outer-membrane domain in a less-flexible conformation were carried out by soaking and co-crystallizing the full-length protein and the isolated HK domain with various additives. The best results were obtained when *Ks*-Amt5 was soaked overnight with the bisindolylmaleimide-based protein kinase C inhibitor Ro 31–8220.

### High-resolution X-ray diffraction analysis

Diffraction data sets were collected at beam line X06SA at the Swiss Light Source (Paul-Scherrer-Institute, Villigen, Switzerland) to a maximum resolution of 1.9 Å. The frames were indexed and integrated using the XDS package^47^ and subsequently scaled to 1.98 Å resolution with AIMLESS (Supplementary Table 2)^48^. The structure of the entire *Ks*-Amt5 trimer was solved by molecular replacement with programs from the CCP4 suite^49^. *A. fulgidus* Amt1 (PBD-ID: 2B2H) was converted to a poly-alanine chain and used as search model in MOLREP^50^. The replacement solution was refined in REFMAC5^51^, and initial electron density maps could easily be improved by correcting the side chain residues in *F*_o_–*F*_c_ difference electron density maps. Further cycles of model building and refinement were done using COOT^52^ and REFMAC5, respectively. The membrane domain of *Ks*-Amt5 was modeled up to amino-acid residue 405. This model was validated using PROCHECK^53^ and MolProbity^54^. For data collection and refinement statistics see Supplementary Table 2. In addition, the histidine kinase TM0853 from *T. maritima*^20^ was also used as search model for molecular replacement in the full-length crystal data, but no solution was obtained.

### Low-resolution X-ray diffraction analysis

Crystals of full-length *Ks*-Amt5 soaked with Ro 31–8220 were harvested and exposed to X-rays in a modified beam line setup that uses a small beam stop near the detector and a helium-filled path between the goniometer and the beam stop to limit air scatter and extend the low-resolution limit to 600 Å^55^. The crystal-detector distance was 700 mm. A complete and highly redundant data set was collected to a maximum resolution limit of 7.5 Å, with 0.25° oscillation range per image and 360° of rotation. After this, the κ angle of the goniometer was changed and a second data set was collected as before. Data sets were integrated and merged with XDS and ab initio low-resolution phasing was performed. The unit cell dimensions for this crystal form were smaller than for the native form, and a difference Fourier synthesis phased from the identified atomic model for the Amt domain revealed additional, non-interpreted electron density assumed to represent the HK domain. This pointed towards a decreased flexibility of the HK domain in the inhibited crystal. To phase the data, four cycles of our connectivity-based phasing procedure were performed^35, 56^. Multiple randomly generated phase sets were tested for connectivity in high-density regions in corresponding Fourier syntheses of the electron density, and suitable phase sets were then averaged, to be used in the next refinement cycle for focusing the search on the vicinity of the found solution. Phasing started from 16 Å resolution (89 reflections) and ended at 8 Å resolution (639 reflections). In the first cycle, the connectivity restriction was to have one large, connected region for every *Ks*-Amt5 molecule. In the next cycles, one large connected domain for Amt and one for HK were permitted, and additionally a number of small, connected “drops”, but the common number of connected regions was restricted. The number of checked random phase sets varied for different cycles from 30 to 3 million. The final 8 Å resolution maps revealed two large high-density regions: one for the Amt domain that fitted the Amt model very well, and one corresponding to the non-modeled HK domain. The map correlation coefficient between the ab initio map and one found by molecular replacement was 73% and 59% for syntheses weighted by figures of merit and unweighted, respectively.

### Construction of homology models for the HK constructs

Homology models for the HK domain were calculated by submitting the corresponding polypeptide sequence to three modeling servers, SWISS-MODEL^57^, I-Tasser^58^, and Phyre2^59^. Both the full-length HK module (residues 401–679) and the truncated construct (residues 426–679) were used as search templates. Default options were used, with the exception of Phyre2, where the *Intensive* modeling mode was used. Results were fit to the low-resolution crystallographic electron density and the SAXS scattering data.

### SAXS

Purified and concentrated *Ks*-Amt5 and *Ks*-Amt5^426–679^ proteins were thawed on ice from liquid nitrogen storage, centrifuged for 10 min at 16,000 × *g* at 4 °C and re-quantified in a UV-visible spectrometer before preparing dilution series of 10, 5, 2, and 1 mg mL^–1^ to accurately estimate the radius of gyration Rg. Protein samples were measured in batch (offline) or in SEC-SAXS (online) mode at beam line P12 (DESY, Hamburg, Germany) using a Superdex 200 10/300 column mounted on site and equilibrated in buffer C supplemented with 5 % (*v/v*) of glycerol and the respective detergent when assaying *Ks*-Amt5, at 20 °C. In order to prevent radiation damage, 2 mM 2-mercaptoethanol or 5 mM DTT were added. To evaluate the effect of ligands on potential conformational rearrangements of the protein, samples were pre-incubated with 2–10-fold molar excess of ligand for 1 h on ice, prior to measurements. Molecular weight estimates were based on forward scattering *I*(0), using water and/or bovine serum albumin as standards. The background scattering contribution from detergent micelles in the protein buffer was negligible in 0.09 % (*w/v*) CYMAL-5 (Anatrace, Suppl Fig. 7). Data reduction and parameter extraction was performed using PRIMUS^60^. Evaluation of the real-space distance distribution function, *p*(r), was performed using GNOM^61^. Subsequent ab initio model building was carried out using DAMMIN^62^ or GASBOR^63^. Analyses of potential conformational transitions in the HK domain were conducted using an elastic network procedure implemented in the program SREFLEX^64^ and homology models for the HK transducing domain as starting points. Modeling of full-length *Ks*-Amt5 was done by several approaches. In the first, the program MEMPROT^65^ was used to build a corona of Cymal-5 dummies around a model based on rigid body positioning of the crystal structure of the transmembrane region and HK domains into the low-resolution crystallography density map. Systematic solutions containing detergent coronas of 220–240 Cymal-5 molecules yielded the best fits to the SAXS data. Following this procedure, an all-atom model of the detergent corona was built using the program PACKMOL^66^ and an input topology file from the Automatic Topology Builder (https://atb.uq.edu.au). The detergent corona was energy minimized in GROMACS 4.5.5^67^ using the GROMOS53A6 force field and incorporated into a hybrid rigid body SAXS modeling procedure in the program CORAL^68^, along with the crystal structure of the membrane domain of *Ks*-Amt5 and homology models of the HK domain. To investigate the potential flexibility of the detergent-solubilized protein, the Ensemble Optimization Method^69^ was also applied using the same input as for CORAL to generate a pool of 10,000 random configurations, and a genetic algorithm employed for selection of ensembles that best fit the scattering data.

### Data availability

The high-resolution coordinates and structure factors for the Amt domain of *Ks*-Amt5 have been deposited with the Protein Data Bank (PDB-ID 6EU6). The SAXS data were deposited with SASBDB (http://sasbdb.org), with accession codes SASDCM9 for *Ks*-Amt5, SASDCN9, SASDCP9, and SASDCQ9 for the histidine kinase, *Ks*-Amt5^426–679^, without ligand, with 10 µM APPCP and 10 µM ATP, respectively. Other data can be made available from the corresponding author upon request.

## Electronic supplementary material

Supplementary information
