# Supplementary material for: Signaling ammonium across membranes through an ammonium sensor histidine kinase
Source: Nat Commun. 2018 Jan 11;9:164. doi: 10.1038/s41467-017-02637-3 (PMC5764959; doi:10.1038/s41467-017-02637-3)
Supplement: Supplementary file 1 — Supplementary information [file 41467_2017_2637_MOESM1_ESM.docx]

**
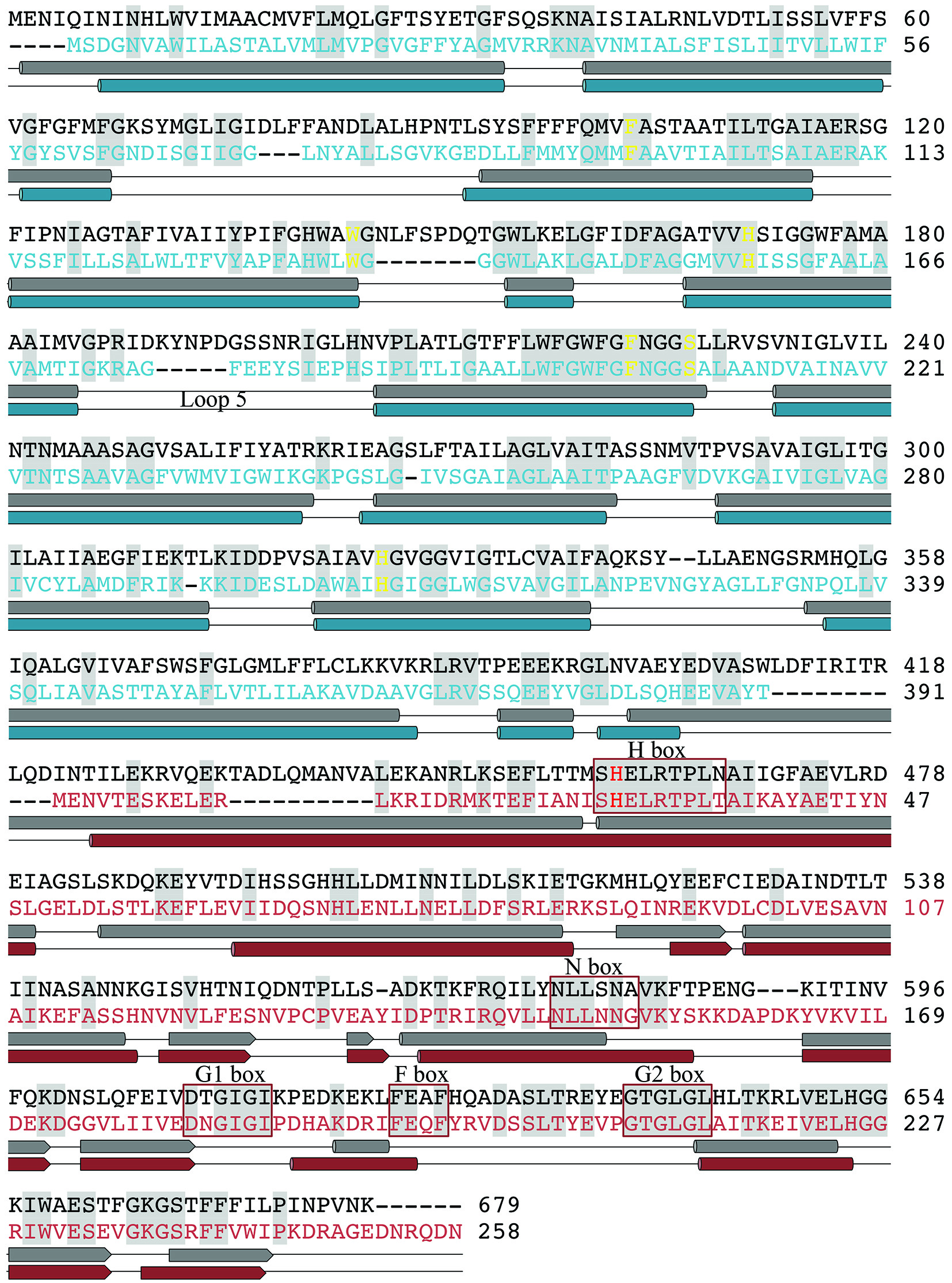
**

**Supplementary Figure 1 (previous page) Amino acid sequence analysis of *Ks*-Amt5.** The N-terminus (residues 1 to 400) of *Ks*-Amt5 (black) is aligned with the assimilatory *Af*-Amt1 (blue). The two polypeptides share 32 % sequence identity. Specific residues, highlighted in yellow, mark the position of conserved and potentially relevant sites for NH_4_^+^ transport in Amt/Rh proteins ^1^. The C-terminal domain of *Ks*-Amt5 (residues 401 to 679, black) displays strong homologies to class I histidine kinases. The HK domain from *Tm*-0853 (red) is the closest homolog, with 33 % sequence identity, while BceS, VanS and DesK share 22, 15 and 14 % sequence identity, respectively. Conserved residues assigned to the H, N, G1 (or D), F and G2 (or G) boxes are shown. The H box harbors the conserved phospho-accepting histidine, H460 (red) and the HisKA subfamily signature, the HE/DxxT/N motif ^2^. Grey shades mark sequence stretches that are 100% identical. Secondary structure elements are derived from the available crystal structures of *Af*-Amt1,^3^ *Ks*-Amt5^1-405^ (this work) and *Tm*-HK0853.^4^ The secondary structural elements for *Ks*-Amt5^401-679^ were predicted using PsiPred.^5^ The alignment was done using Clustal Omega.^6^


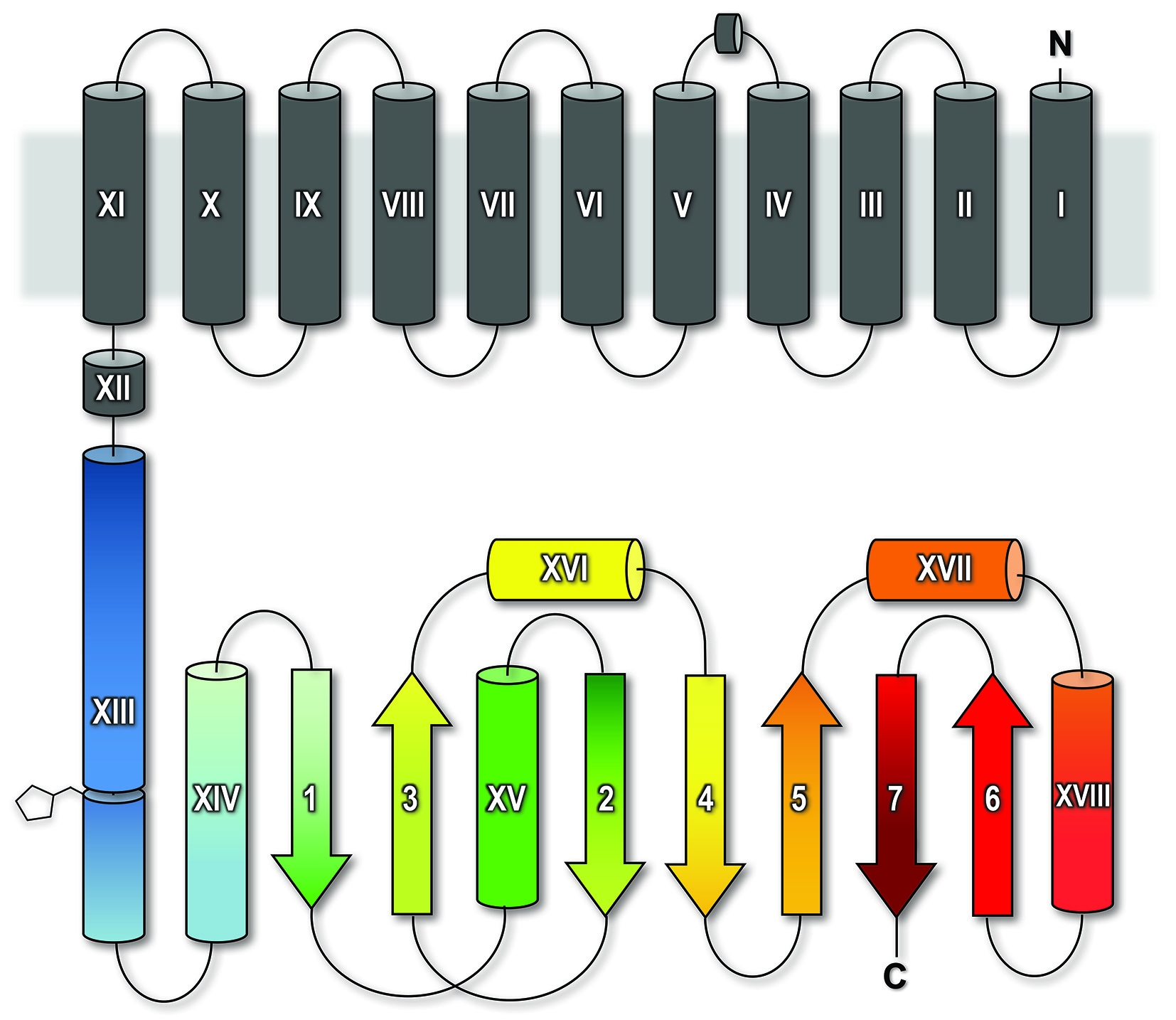


**Supplementary Figure 2 Predicted secondary structure and topology of *Ks*-Amt5.** The N-terminal Amt domain is shown in grey and comprises transmembrane helices *h*I to *h*XI followed by a short linker of about 17 amino acid residues (K384 – N400) containing a short helical segment of 6 amino acids (outer-membrane *h*XII). Immediately following this, the HK domain starts (colored from blue to red). Helices *h*XIII and *h*XIV form the dimerization and histidine phosphotransfer (DHp) domain harboring the putative functional His460 (side chain depicted as sticks). The following residues are predicted to form the globular catalytic and ATP-binding (CA) domain. Secondary structure elements were predicted using PsiPred.^5^

**
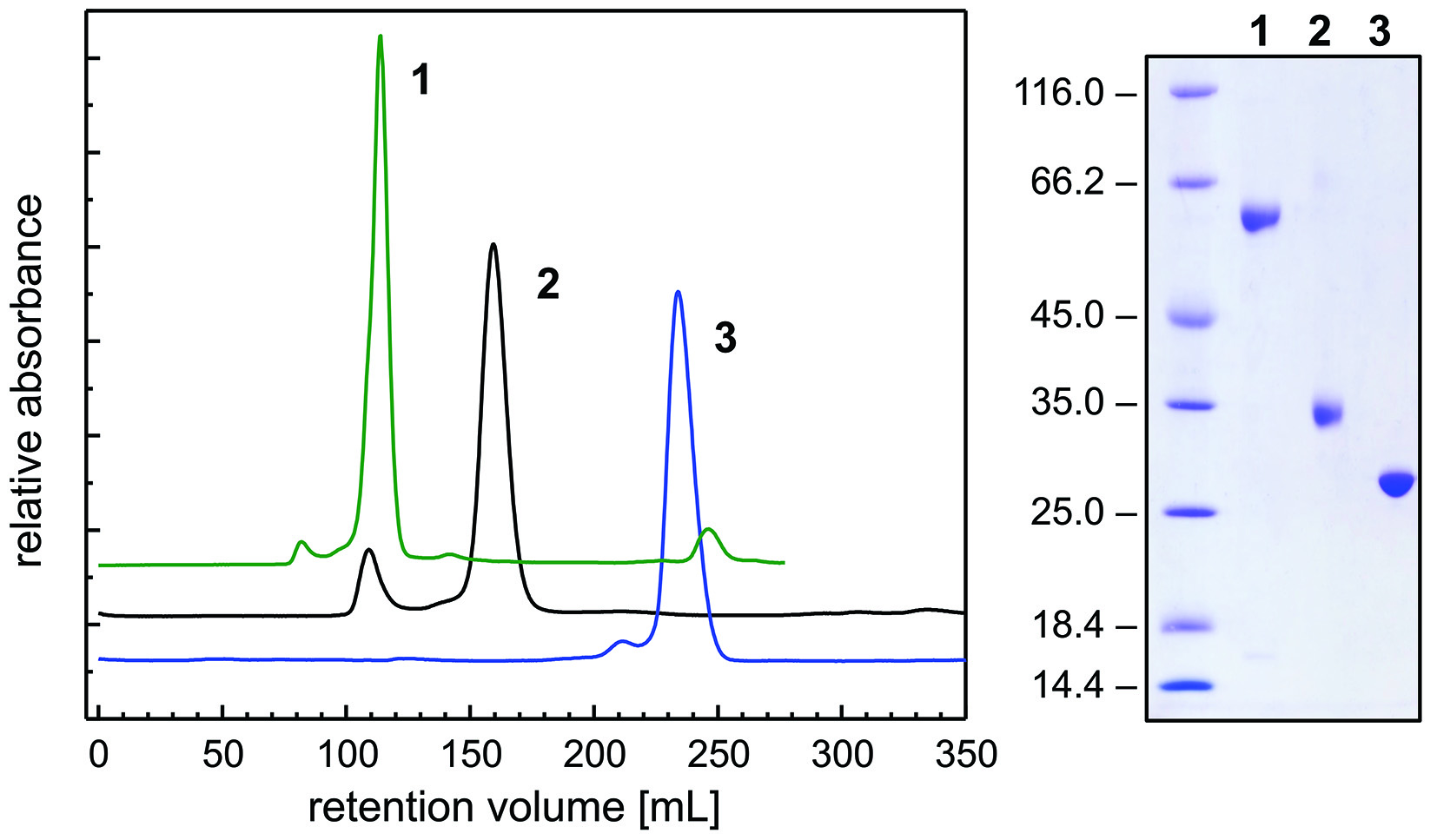
**

**Supplementary Figure 3 Purity and oligomeric state assessment of isolated *Ks*-Amt5 and its individual modules.** Size-exclusion chromatograms (left) of *Ks*-Amt5 (**1**) and *Ks*-Amt5^1-408^ (**2**) confirm that the proteins are trimeric in detergent solution. In contrast, the retention volume of *Ks*-Amt5^426-679^ (**3**) corresponds to a monomeric species. The high purity level of all samples is noticeable on a 10 % SDS-PAGE after Coomassie staining that shows the molecular weight marker (Thermo Fisher) and the individual, monomeric protein bands.

**
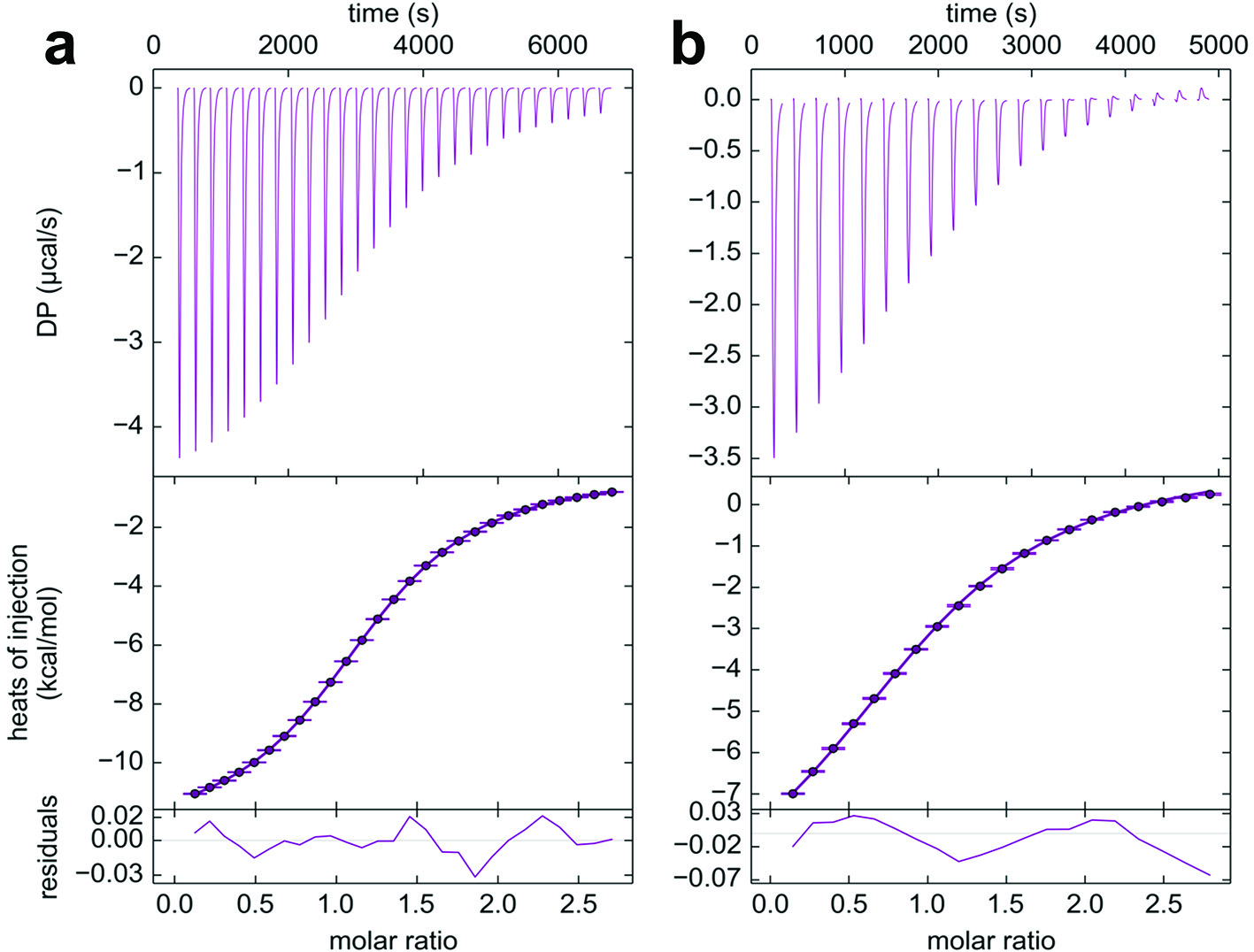
**

**Supplementary Figure 4 Thermodynamic analysis of nucleotide binding to *Ks*-Amt5^426-679^**. The heat developed during the titration of the outer-membrane domain with Mg-ATP (**a**) and the non-hydrolysable analog Mg-ATPγS (**b**) was integrated and the data points were fitted with a single-site-binding model (below). The results revealed dissociation constants for ATP / ATPγS of 11.16 ± 0.03 / 36.74 ± 0.05 μM, enthalpy changes of ­–14.98 / –11.92 kcal·mol^–1^ and entropy changes of –27.58 / –20.36 kcal·mol^–1^·K^–1^. In contrast, the protein kinase C inhibitor Ro 31-8220, Mg-GTP, ADP and AMP did not bind to the protein and a variant carrying bulkier residues in the nucleotide-binding site (I612W-S666W) was, as expected, impaired in nucleotide binding.

**
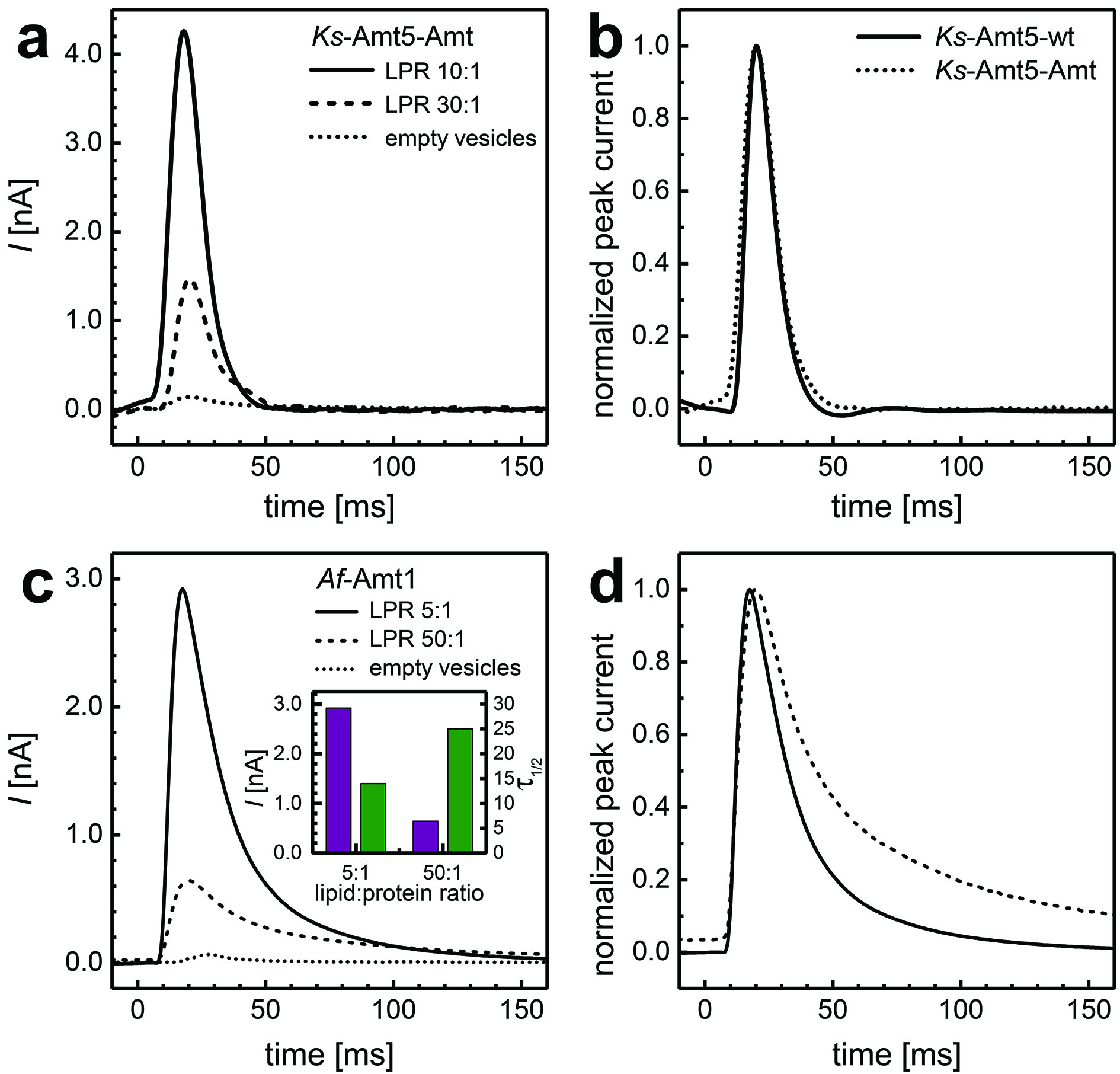
**

**Supplementary Figure 5 Electrogenic transport characteristics of the *Ks*-Amt5^1-408^ domain in proteoliposomes.** (**a**) Transient currents for the isolated Amt domain reconsti­tuted in vesicles at LPR 10:1 (solid), LPR 30:1 (dashed) and for empty vesicles (dotted). The response strongly resembles that for full-length *Ks*-Amt5 (b). The half-maximum decay time was nearly independent of changes in LPR (τ_½_^LPR 10:1^ = 7.7; τ_½_^LPR 30:1^ = 8.8 ms, Fig. 2b), pointing towards charge displacement rather than charge translocation. (**b**) Normalized peak currents for full-length protein (solid) and Amt domain (dotted), reconstituted at LPR 10:1 reveal identical transients with half-maximum decay times of 8.5 and 9.0 ms, respectively. Data recorded with concentration jumps of 300 mM NH_4_^+^ at pH 7.5. (**c**) Reference transients for a typical, assimilatory Amt protein, Amt-1 from *A. fulgidus*.^7^ Note that both peak currents (inset, purple) and τ_½_ (inset, green) change markedly at different LPR, indicating true charge translocation. (**d**) Norma­li­za­tion of the currents shown in (c), highlighting the extended decay times at LPR 50:1.


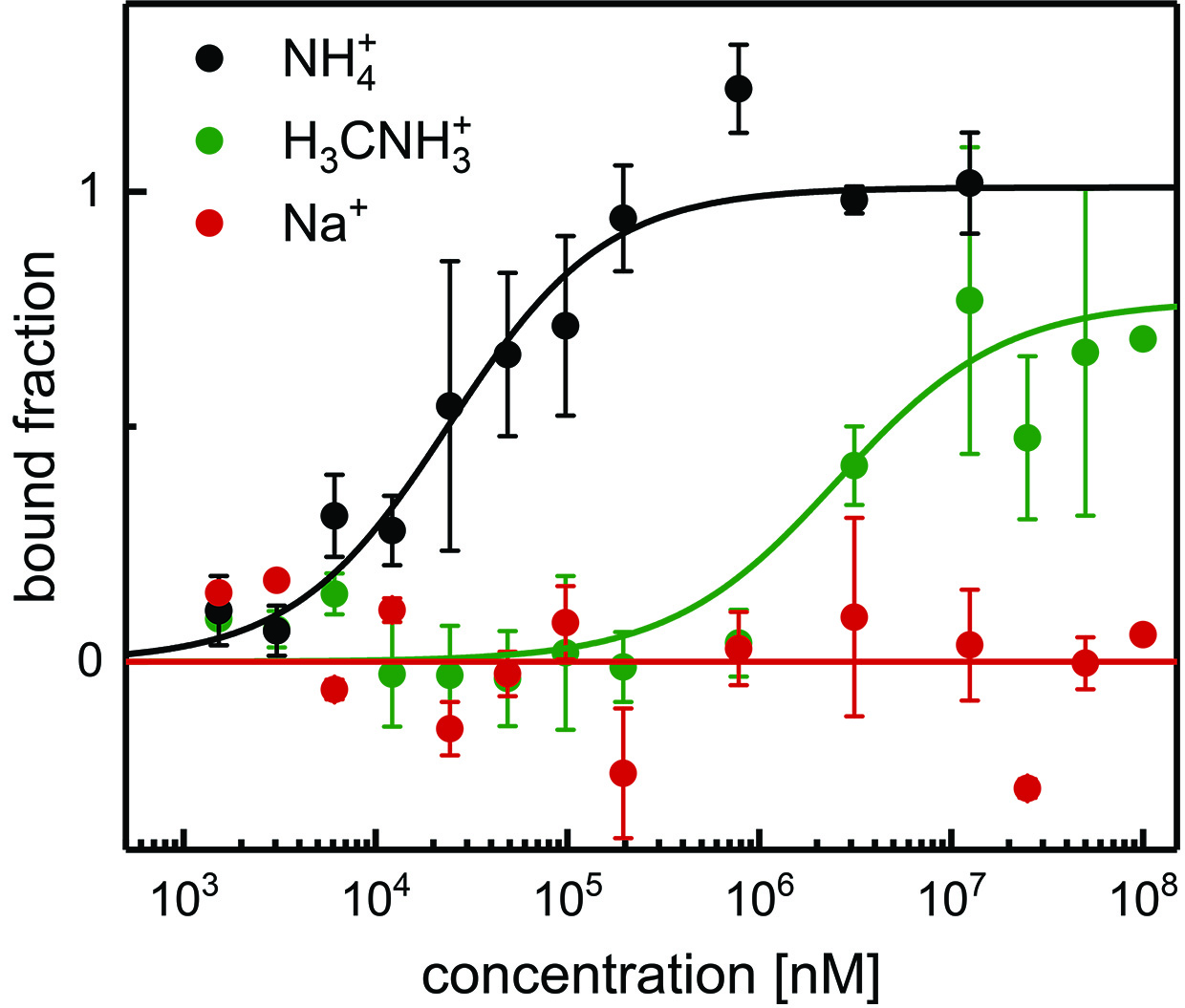


**Supplementary Figure 6 Characterization of ion binding to full-length *Ks*-Amt5 by microscale thermophoresis.** Ammonium (black), MA (green) and a non-substrate control cation (Na^+^, red) were used for thermophoresis at a protein concentration of 200-500 nM. The *K_d_* value determined from a sigmoidal fit to the data collected in triplicates was *K_d_* = 41 ± 24 µM for ammonium, but two orders of magnitude higher for methylammonium, with *K_d_* = 2350 ± 440 µM. Confirming the results from SSM-based electrophysiology, Na^+^ did not show any binding to the protein.

**
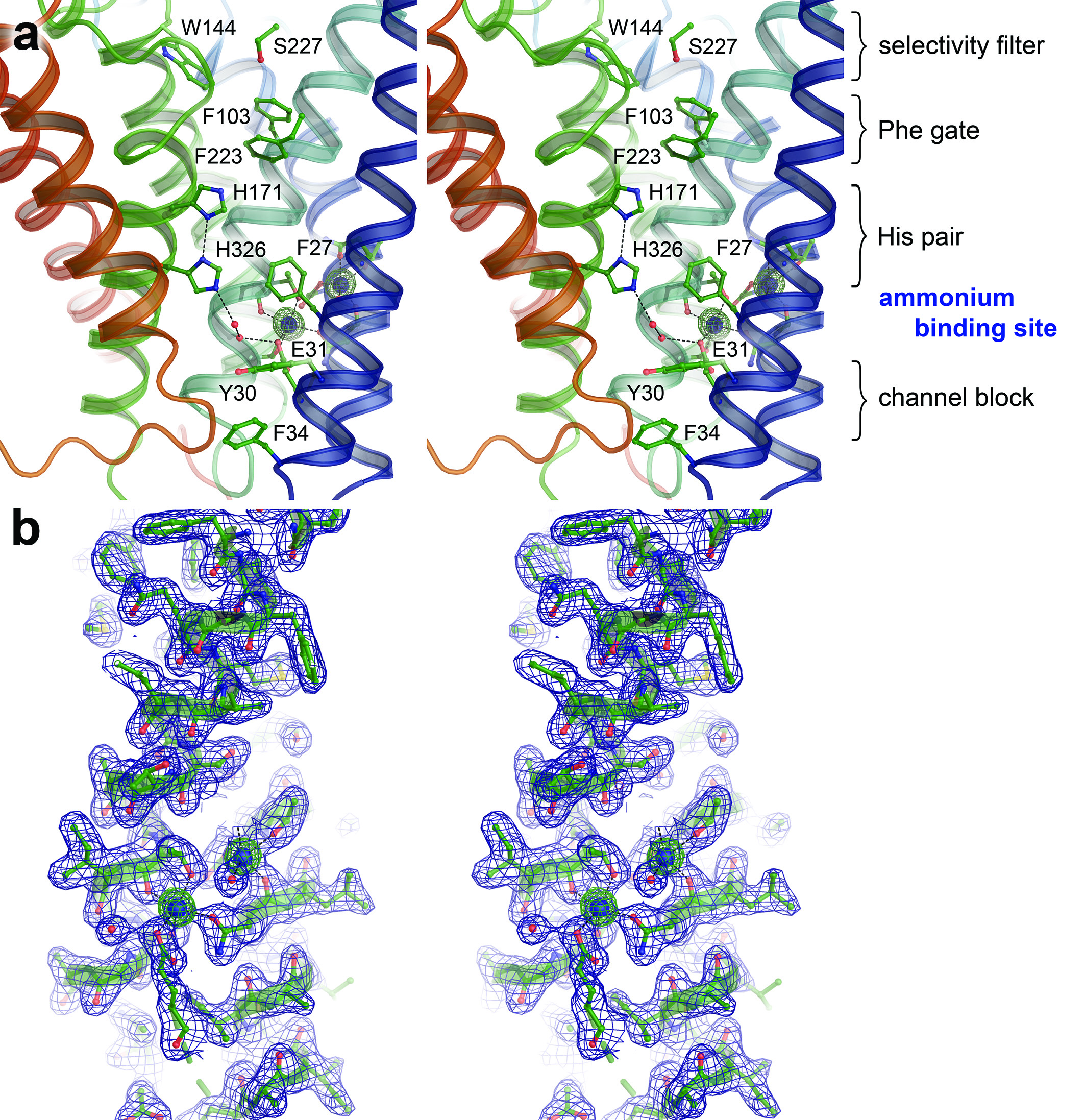
**

**Supplementary Figure 7 Structural context of the substrate channel and the cation binding sites in *Ks*-Amt5. (a)** The Amt domain of the sensor retains the conserved structural features of the ammonium transporter family.^8^ These include a selectivity filter (W144/S227) that selects for substrates able to engage in cation-π interaction with tryptophan as well as H-bonding to the serine residue, as well as a phenylalanine pair (‘Phe gate’, F103/F223) that presumably must rearrange for NH_4_^+^ ions to pass, effectively removing their hydration shell. Below the Phe gate, H171 and H326 form the characteristic ‘His pair’ that is highly conserved in Amt proteins. Close to H326, however, the pathway to the cytoplasm is obstructed by Y30 and F34, while residue F27 replaces a bulkier tryptophan found in assimilatory Amt proteins. This provides direct access to the nearby cation binding site, so that *Ks*-Amt5 retains all features required to select for NH_4_^+^ cations and guide them into the membrane, but diverts the ions away from the exit channel into the novel holding site of the sensor protein. In the stereo image, the protein is displayed in cartoon representation colored from blue at the N-terminus to red at the C-terminus. TM helices VI-IX are omitted for clarity. For the cations in the ammonium binding site, a *F*_o_–*F*_c_ omit difference electron density map contoured at the 5.0 σ level is shown in green. The representation shows the N-terminal side at the top and the C-terminal side at the bottom. **(b)** Stereo visualization of a representative segment of the 2*F*_o_–*F*_c_ electron density map contoured at the 1 σ level. The region shown comprises transmembrane helices II and III, as well as NH_4_^+^ binding site.


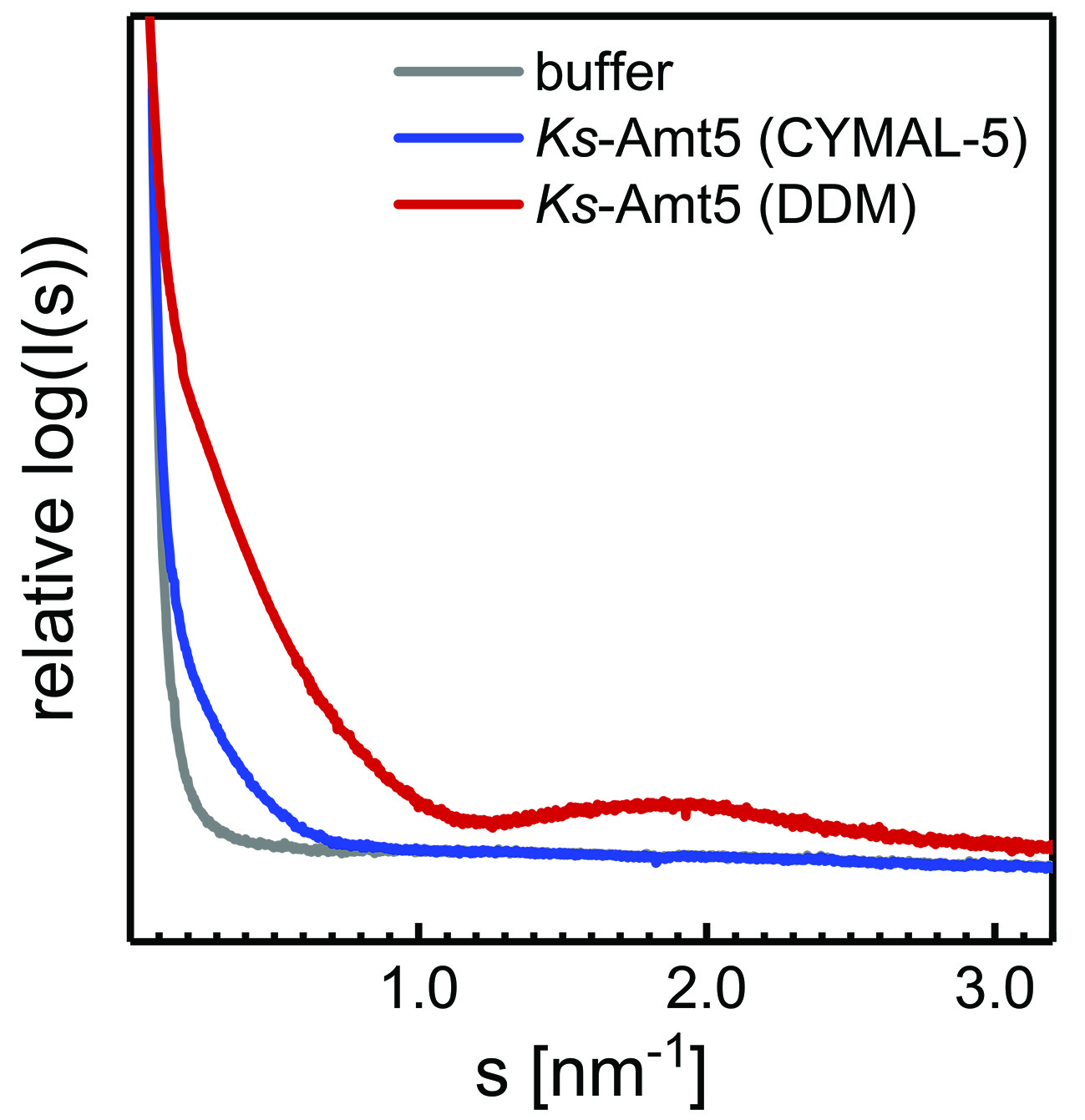


**Supplementary Figure 8 Small-angle scattering behavior of *Ks*-Amt5 in CYMAL-5 *vs*. DDM.** *Ks*-Amt5 was originally extracted and crystallized in maltosides (0.03 % (*w/v*) DDM and a mixture of 0.03 % DDM and 0.65 % NM, respectively), but in initial SAXS studies these preparations (red) showed substantial background scattering above that of the buffer (grey), with features around *s* = 2 nm^–1^ that are typical for unwanted detergent micelles. This was very well alleviated by detergent exchange into 0.09 % of CYMAL-5 (blue), and these preparations were used for all subsequent SAXS measurements.


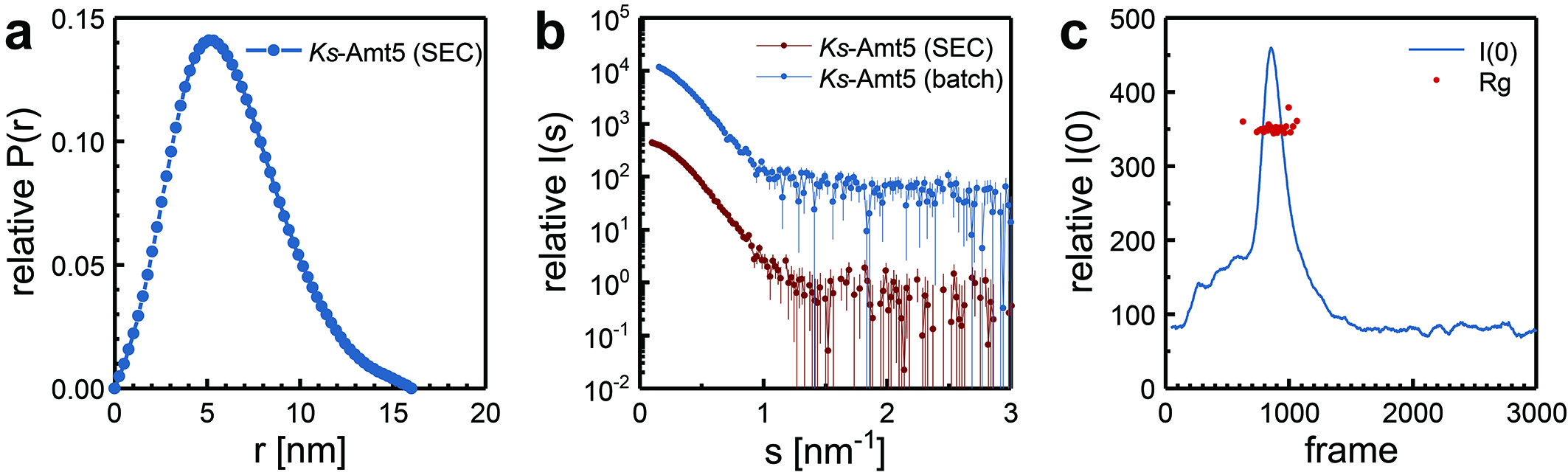


**Supplementary Figure 9 Small-angle X-ray scattering (SAXS) analysis of full-length *Ks*-Amt5 solubilized in 0.09 % (w/v) CYMAL-5.** (**a**) Real space distance distribution *p(r)* for the SEC-SAXS measurements. (**b**) Experimental scattering records of batch (blue) and SEC-SAXS (red) samples. (**c**) SEC-SAXS trace with forward scattering intensity, *I(0)* (blue line) and radius of gyration, R*g* (red) across the major peak.

**
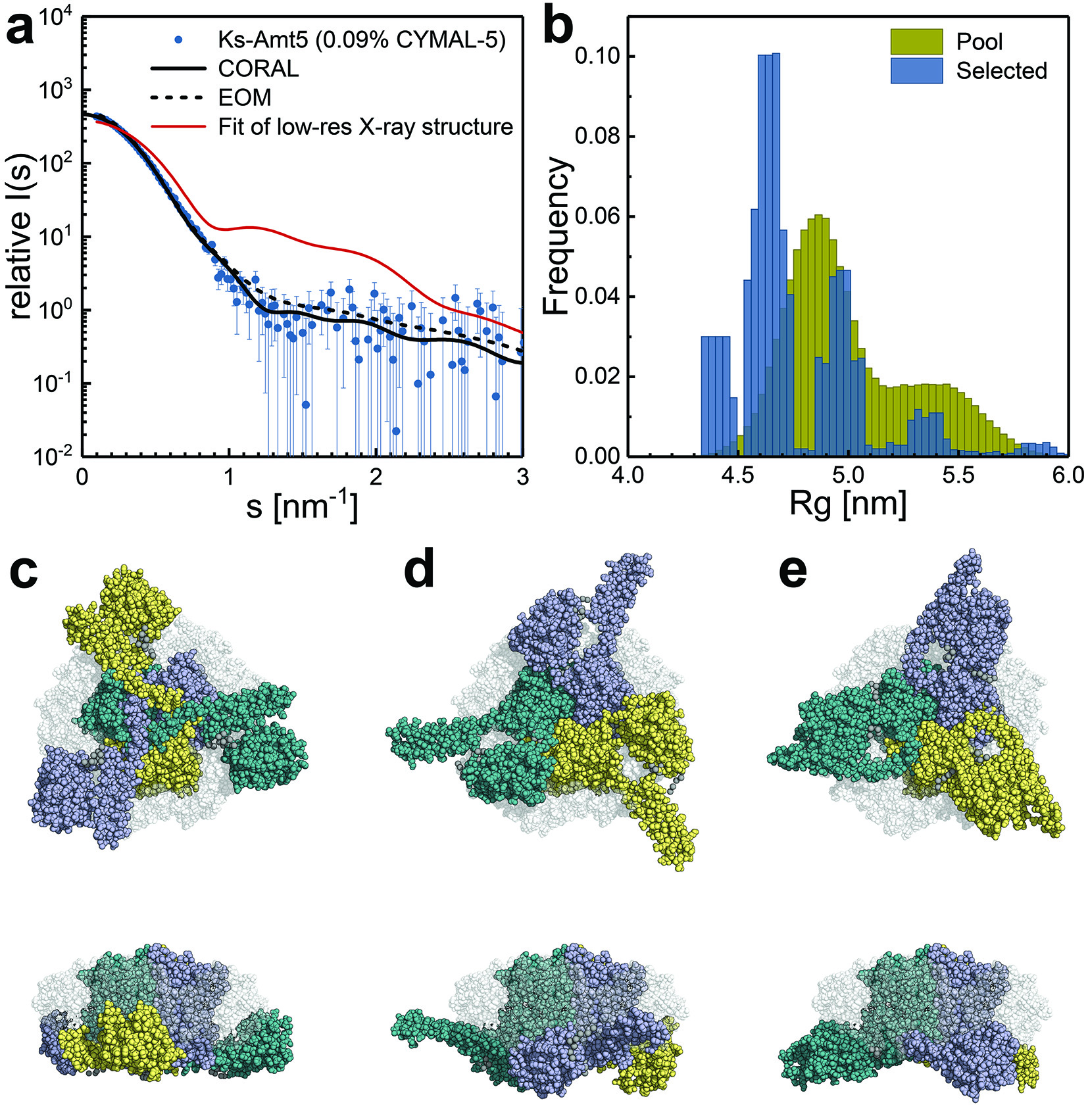
**

**Supplementary Figure 10 Modelling and flexibility analysis of *Ks*-Amt5.** (**a**) SAXS data for protein solubilized in CYMAL-5 (blue circles) and fits of the best rigid body model from CORAL (solid black line) and of the EOM ensemble (dotted black line). Notably, the predicted SAXS from the low-resolution volume obtained by X-ray diffraction does not fit the experimental data, emphasizing that the crystal packing restricts conformational flexibility on the kinase domains, which is not the case in solution. (**b**) Distribution of radius of gyration from the EOM analysis. The selected ensemble (blue) and random pool (olive) are shown. (**c–e**) Representative rigid body models of *Ks*-Amt5 generated using the program CORAL. Each monomer of the trimeric protein is shown as spheres of a single color (grey, green or yellow) and the detergent belt is shown in white.

**
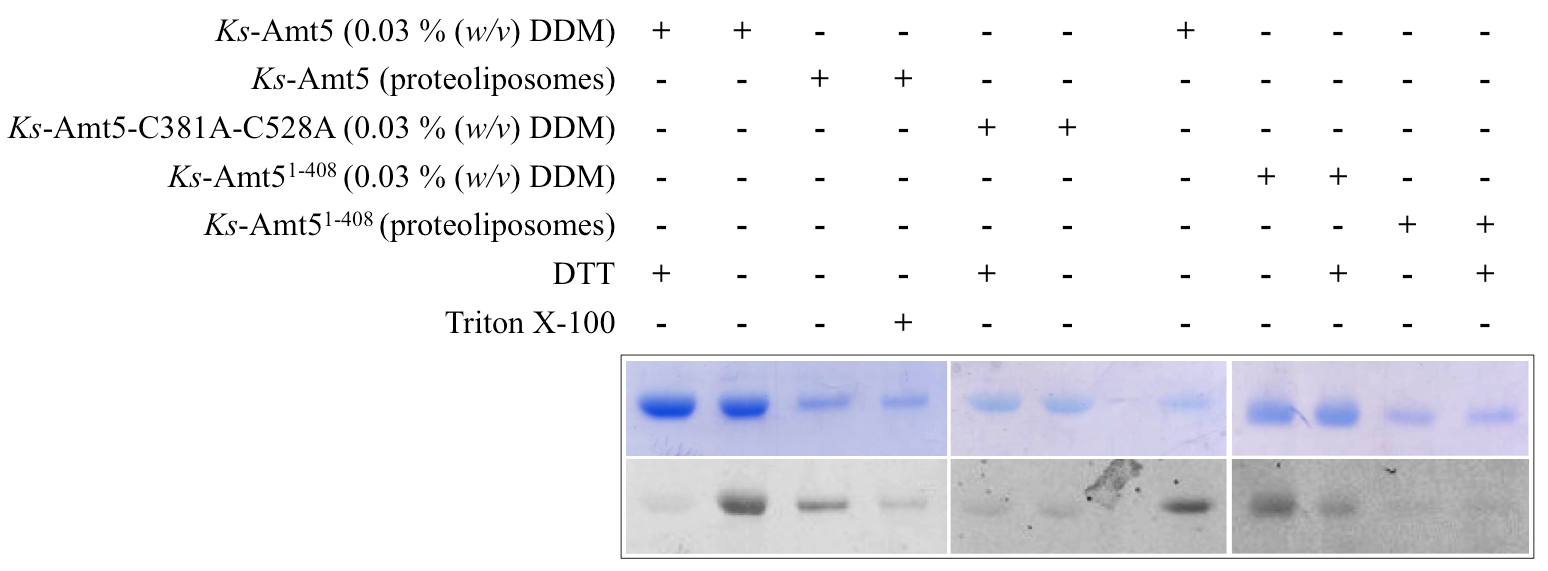
**

**Supplementary Figure 11 Orientation of *Ks*-Amt5 in proteoliposomes.** A fluorescence-based assay targeted exposed cysteine residues in the protein after a reaction with tetramethyl-rhodamine-5-maleimide (TMR). Dithiothreitol (DTT) was used to prevent (if added before TMR (+)) or to stop the reaction with TMR. Reaction mixtures were analyzed in 10 % SDS-PAGE gels that were imaged under UV light (down) and further stained with Coomassie brilliant blue (top). To destabilize the vesicles and potentially increase the exposure of the single outer-membrane cysteine residue (C528 in the CA domain of the HK module) to TMR, we added Triton X-100. Under these conditions, the decrease in band intensity revealed that the majority of proteins inserted with the HK module facing the outside medium. This conclusion is confirmed by the action of DTT, where a strong decrease in the UV-light band intensity was only observed when DTT was added to the full-length protein. The effect of DTT was severely diminished when testing protein constructs that only have cysteine residues in the membrane module (C18, C336 and C381 in *Ks*-Amt5^1-408^ or C18 and C336 in *Ks*-Amt5-C381A-C528A) and always produce a residual UV-light signal.

**
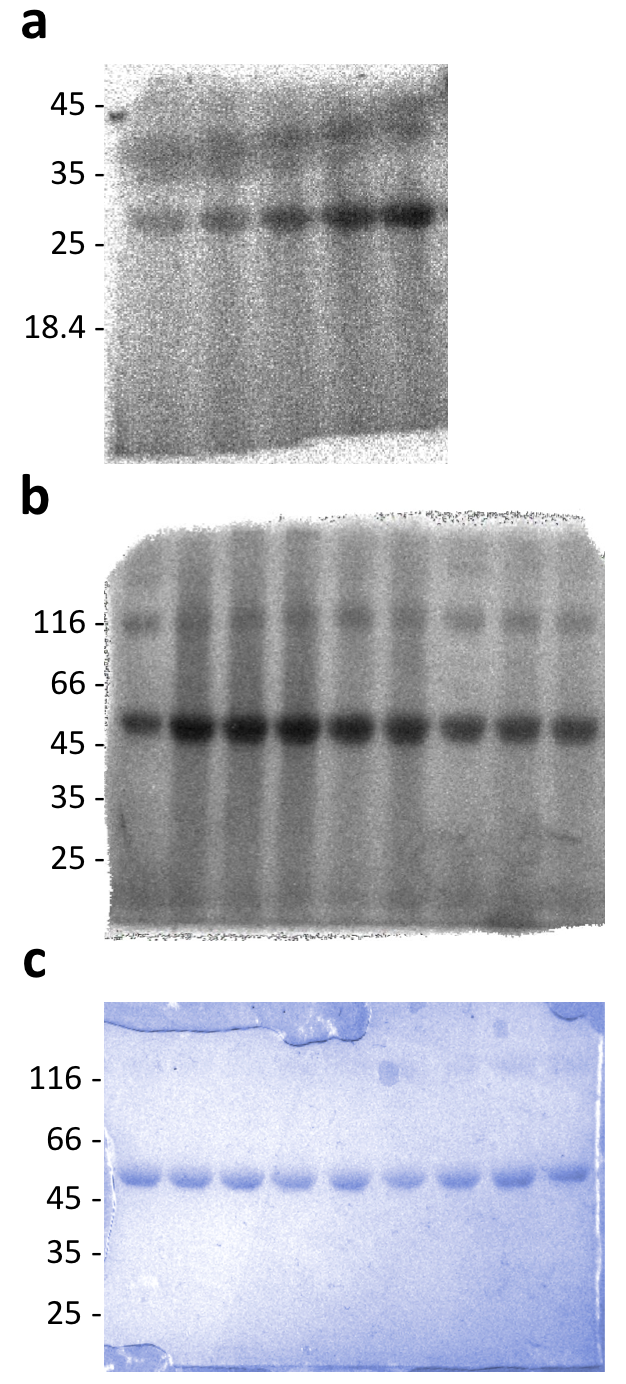
**

**Supplementary Figure 12 Original blots and SDS-PAGE gels depicted in figure 1.** Autoradiographies of **(a)** purified HK domain incubated with γ-^32^P-ATP for 5, 10, 20, 40 and 60 min (left to right) and **(b)** purified full-length *Ks*-Amt5 incubated for 60 min with γ-^32^P-ATP as a function of increasing ammonium concentrations (from left to right: 0, 5, 10, 20, 50, 100, 200, 300 and 400 mM NH_4_Cl). Under these denaturing conditions, the observed darker and lighter protein bands correspond to the dominant monomeric and trimeric phosphorylated *Ks*-Amt5. **(c)** Coomassie-stained SDS-PAGE of (b).

**
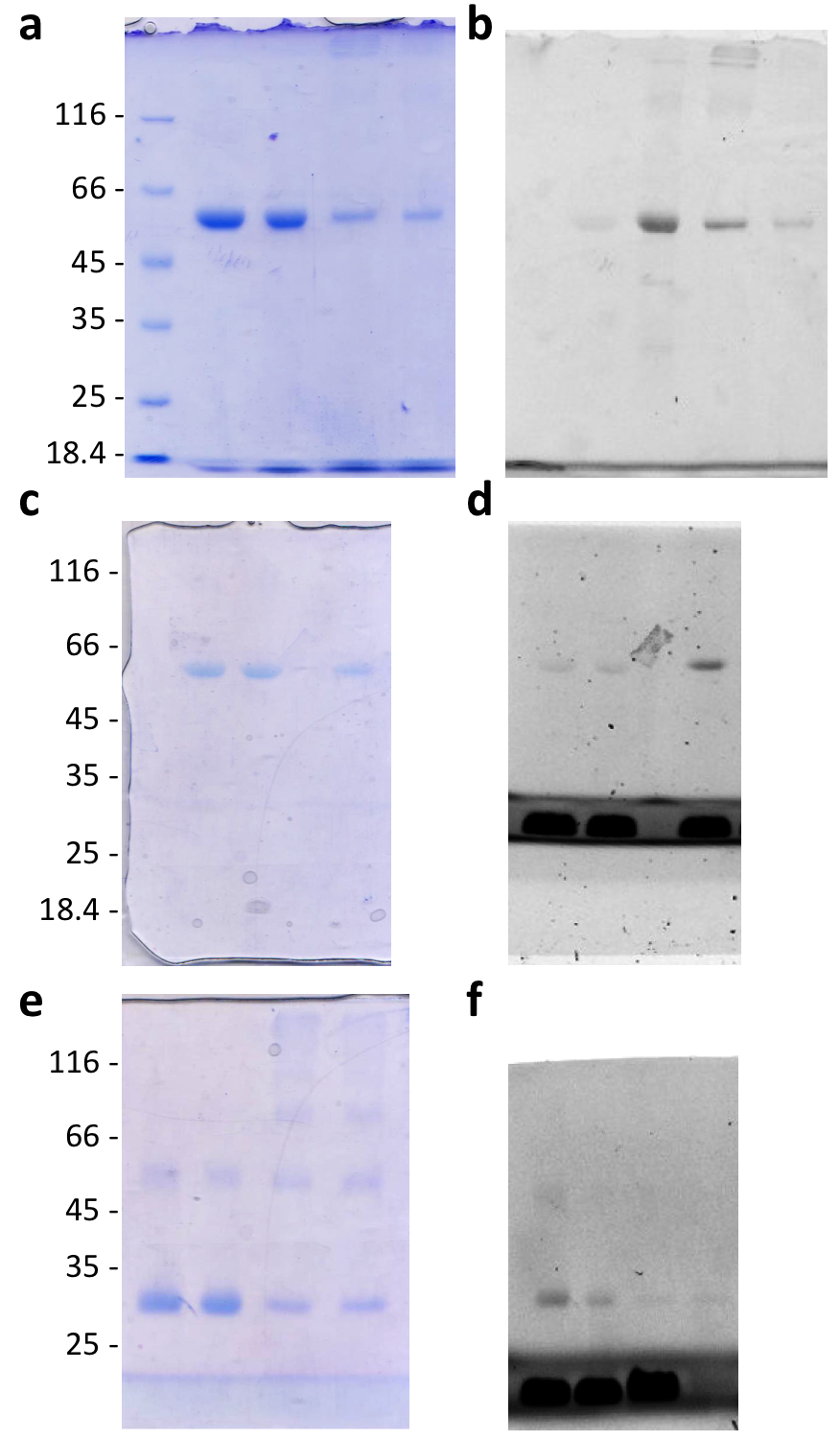
**

**Supplementary Figure 13 Original SDS-PAGE gels depicted in supplemental figure 11. (a)** Lane 1 – molecular weight markers (116, 66.2, 45, 35, 25 and 18.4 kDa); lane 2 – pure *Ks*-Amt5 in 0.03 % (w/v) DDM with and without (lane 3) DTT; lane 4 and 5 – *Ks*-Amt5 reconstituted in proteoliposomes, undisrupted (lane 4) and disrupted (lane 5) using Triton X-100. Gel was stained with Coomassie. **(b)** Same as (a), but imaged with UV light. **(c)** Lane 1 – Isolated *Ks*-Amt5 C381A-C528A variant prepared in 0.03 % (w/v) DDM and incubated with DTT; Lane 2 – same as lane 1 without DTT; Lane 3 – empty; Lane 4 – Isolated wild-type *Ks*-Amt5 in 0.03 % (w/v) DDM as reference. Gel was stained with Coomassie. **(d)** Same as (c) but, imaged with UV light. **(e)** Lane 1 – Isolated Amt domain of *Ks*-Amt5 in 0.03 % (w/v) DDM; Lane 2 – same as Lane 1 after treatment with DTT; Lane 3 – Amt domain of *Ks*-Amt5 reconstituted into proteoliposomes; Lane 4 – same as lane 3 incubated with DTT. Gel was stained with Coomassie. **(f)** Same as (e), but imaged with UV light.

**Supplementary Table 1 DNA primer sequences used to produce recombinant *Ks*-Amt5 and variants.** Restriction sites are underlined. Point mutations are shown in bold.

| Construct | Primer^a^ | Oligonucleotide sequences (5’ – 3’) |
| --- | --- | --- |
| pCR2.1-TOPO::*amt5*^1-679^ | F1 | GGATCCATGGAAAACATACAAATAAATATTAAC |
|  | R1 | CTTGCCCATAAATCCAGTGAACAAGGGTACC |
| pET21a::*amt5^1-679^* | F2 | AGATATACATATGGAAAACATACAAAT |
|  | R2 | CCATAAATCCAGTGAACAAGCTCGAGAAT |
| pET21a::*amt5^1^*^-408^ | R3 TGTCGCCGAATATGAAGACGTTGCGCACCACCACCACCACCACTGAGATC | |
| pET15dt::*amt5^426-679^* | F3 | AAATACACATATGCTTGAAAAAAGGGT |
|  | R4 | GCCCATAAATCCAGTGAACAAGCTCTCGAGAAT |
| H460A | F4^b^ | CAACAATGTCA**GCT**GAGCTGCGCAC |
| S666W | F5^b^ | CCTTTGGAAAAGGA**TGG**ACCTTCTTTTTTATCTTGC |
| I612W | F6^b^ | GATACCGGCATTGGT**TGG**AAGCCTGAAGACAAAG |
| C381A | F7^b^ | GCTTTTCTTCTTG**GCG**CTAAAGAAAG |
| C528A | F8^b^ | CGAGGAATTT**GCG**ATTGAAGATGC |

^(a)^ F: forward; R: reverse. ^(b)^ For site-directed mutagenesis, only the forward oligonucleotide primer sequence is provided.

**Supplementary Table 2** **Data collection and refinement statistics for *Ks*-Amt5 (PDB-ID 6EU6).**

|  | Native |
| --- | --- |
| **Data collection** |  |
| Space group | *P*6_3_ |
| Cell dimensions |  |
| *a*, *b*, *c* (Å) | 99.8, 99.8, 89.1 |
| α, β, γ (°) | 90.0, 90.0, 120.0 |
|  |  |
| Wavelength (Å) | 1.00000 |
| Resolution (Å) | 86.38 – 1.98 (2.03 – 1.98) |
| *R*_merge_ | 0.112 (1.461) |
| *R*_p.i.m._^9^ | 0.037 (0.469) |
| *CC*_(1/2)_^10^ | 0.999 (0.735) |
| *I* / σ*I* | 12.1 (2.2) |
| Completeness (%) | 100.0 (99.9) |
| Redundancy | 10.3 (10.5) |
|  |  |
| **Refinement** |  |
| Resolution (Å) | 86.38 – 1.98 |
| No. reflections | 33,401 (2,466) |
| Figure of merit | 0.868 |
| *R*_work_ / *R*_free_ | 0.154 / 0.185 |
| No. atoms |  |
| Protein | 3,078 |
| Water | 107 |
| *B*-factors (Å^2^) |  |
| Protein | 36.65 |
| Ligand | 60.63 |
| Water | 43.04 |
| R.m.s deviations |  |
| Bond lengths (Å) | 0.019 |
| Bond angles (°) | 1.964 |

* values in parentheses are for highest-resolution shell.

**Supplementary Table 3 Low-resolution diffraction data statistics for *Ks*-Amt5 soaked with Ro 31-8220.**

|  | Native |
| --- | --- |
| **Data collection** |  |
| Space group | *P*6_3_ |
| Cell dimensions |  |
| *a*, *b*, *c* (Å) | 105.0, 105.0, 91.17 |
| α, β, γ (°) | 90.0, 90.0, 120.0 |
|  |  |
| Wavelength (Å) | 0.97200 |
| Resolution (Å) | 91.17 – 7.50 (8.39 – 7.50) |
| *R*_merge_ | 0.071 (0.344) |
| *R*_p.i.m._^9^ | 0.011 (0.075) |
| *CC*_(1/2)_^10^ | 1.000 (0.995) |
| *I* / σ*I* | 34.9 (13.4) |
| Completeness (%) | 100.0 (100.0) |
| Unique reflections | 769 (210) |
| Redundancy | 41.0 (42.3) |

* values in parentheses are for highest-resolution shell.

**Supplementary Table 4 SAXS data collection and derived parameters for *Ks*-Amt5.** Protein obtained from batch purification or immediately after SEC-SAXS, in 0.09% CYMAL-5. Abbreviations: *M_r_*: molecular mass; *R_g_*: radius of gyration; *D_max_*: maximal particle dimension; *V_p_*: Porod volume, *M_r_* = *V_p_*/1.7 ; *V_ex_*: Particle excluded volume, *M_r_* = *V_ex_*/2.

|  | from batch | from SEC-SAXS |
| --- | --- | --- |
| **Data collection** |  |  |
| Beam geometry | 0.2 × 0.12 mm^2^ | 0.2 × 0.12 mm^2^ |
| Wavelength (Å) | 1.24 | 1.24 |
| *s* range (Å^-1^)^a^ | 0.01 – 0.46 | 0.01 – 0.46 |
| Exposure time (s) | 1 (20 × 0.05) | 1 (1 × 1.0) |
| Concentration range (mg∙mL^–1^) | ~ 0.1 | ~ 0.1 |
| Temperature (K) | 293 | 293 |
|  |  |  |
| **Structural parameters^b^** |  |  |
| *I*(0) (relative) (from *p*(*r*)) | 14,060 ± 100 | 473 ± 2 |
| *R_g_* (Å) (from *p*(*r*)) | 48 ± 1 | 49 ± 1 |
| *I*(0) (relative) (from Guinier) | 14,318 ± 160 | 470 ± 2 |
| *R_g_* (Å) (from Guinier) | 49 ± 1 | 48 ± 1 |
| *d*_max_ (Å) | 170 | 170 |
| Porod volume (*V_p_*) estimate (Å^3^) | 481,810 ± 50,000 | 510,930 ± 50,000 |
| Excluded volume (*V_ex_*) est. (Å^3^) | 543,600 ± 50,000 | 557,800 ± 50,000 |
| Dry volume from sequence | 95,227 / 285,682 | (monomer / trimer) |
|  |  |  |
| **Molecular mass determination** |  |  |
| *I*(0) (relative) BSA (72,000 Da) | 4,361 ± 5 | n. d. |
| *M_r_* (Da) from *I*(0) | 236,390 ± 30,000 | n. d. |
| *M_r_* (Da) from Porod volume | 283,418 ± 30,000 | 300,547 ± 30,000 |
| *M_r_* (Da) from from excl. volume | 271,800 ± 30,000 | 278,900 ± 30,000 |
| Calc. monomer *M_r_* from sequence | 75,515 | 75,515 |
|  |  |  |
| **Software employed** |  |  |
| Primary data reduction | RADAVER | |
| Data processing | PRIMUS / Qt | |
| *Ab initio* analysis | DAMMIF / GASBOR | |
| Validation and averaging | DAMAVER | |
| Molecular Dynamics | GROMACS | |
| Equilibrium analysis | EOM | |
| Computation of model intensities | CRYSOL | |
| 3D graphics representation | PyMOL / UCSF Chimera | |

^a^ Momentum transfer |*s*| = 4πsin(θ)/λ. ^b^ Values reported for averaged data sets (*Ks*-Amt5 batch: 11 peak fractions ~0.1 mg∙mL^–1^, SEC-SAXS: 40 frames across peak ~0.1 mg∙mL^–1^)

**Supplementary Table 5** **SAXS data collection and derived parameters for *Ks*-Amt5^426-679^.** Abbreviations: *M_r_*: molecular mass; *R_g_*: radius of gyration; *D_max_*: maximal particle dimension; *V_p_*: Porod volume, *M_r_* = *V_p_*/1.7 ; *V_ex_*: Particle excluded volume, *M_r_* = *V_ex_*/2.

|  | APO | + APPCP | | +ATP |
| --- | --- | --- | --- | --- |
| **Data collection** |  |  | |  |
| Beam geometry | 0.2 × 0.12 mm^2^ | 0.2 × 0.12 mm^2^ | | 0.2 × 0.12 mm^2^ |
| Wavelength (Å) | 1.24 | 1.24 | | 1.24 |
| *s* range (Å^-1^)^a^ | 0.01 – 0.46 | 0.01 – 0.46 | | 0.01 – 0.46 |
| Exposure time (s) | 1 (20 × 0.05) | 1 (20 × 0.05) | | 1 (20 × 0.05) |
| Concentration range (mg∙mL^–1^) | 1.0 – 5.0 | 1.0 – 5.0 | | 1.0 – 5.0 |
| Temperature (K) | 293 | 293 | | 293 |
|  |  |  | |  |
| **Structural parameters^b^** |  |  | |  |
| *I*(0) (relative) (from *p*(*r*)) | 2,065 ± 7 | 1,975 ± 7 | | 1,801 ± 6 |
| *R_g_* (Å) (from *p*(*r*)) | 28 ± 1 | 23 ± 1 | | 22 ± 1 |
| *I*(0) (relative) (from Guinier) | 2,046 ± 9 | 1,976 ± 5 | | 1,795 ± 7 |
| *R_g_* (Å) (from Guinier) | 27 ± 1 | 23 ± 1 | | 22 ± 1 |
| *d*_max_ (Å) | 100 | 80 | | 77 |
| Porod volume (*V_p_*) estimate (Å^3^) | 71,000 ± 5,000 | 53,000 ± 5,000 | | 53,000 ± 5,000 |
| Excluded volume (*V_ex_*) est. (Å^3^) | 87,000 ± 10,000 | 65,000 ± 5,000 | | 64,000 ± 5,000 |
| Dry volume from sequence | 34,892 | 34,892 | | 34,892 |
|  |  |  | |  |
| **Molecular mass determination** |  |  | |  |
| *I*(0) (relative) BSA (72,000 Da) | 4,519 ± 5 | 4,519 ± 5 | | 4,519 ± 5 |
| *M_r_* (Da) from *I*(0) | 32,598 ± 3,000 | 31,478 ± 3,000 | | 28,605 ± 3,000 |
| *M_r_* (Da) from Porod volume | 42,000 ± 5,000 | 31,000 ± 3,000 | | 31,000 ± 3,000 |
| *M_r_* (Da) from from excl. volume | 43,000 ± 5,000 | 32,000 ± 3,000 | | 32,000 ± 3,000 |
| Calc. monomer *M_r_* from sequence | 28,836 | 28,836 | | 28,836 |
|  |  |  | |  |
| **Software employed** |  |  | |  |
| Primary data reduction | RADAVER | |  | |
| Data processing | PRIMUS / Qt | |  | |
| *Ab initio* analysis | DAMMIF / GASBOR | |  | |
| Validation and averaging | DAMAVER | |  | |
| Molecular Dynamics | GROMACS | |  | |
| Equilibrium analysis | EOM | |  | |
| Computation of model intensities | CRYSOL | |  | |
| 3D graphics representation | PyMOL / UCSF Chimera | |  | |

^a^ Momentum transfer |*s*| = 4πsin(θ)/λ. ^b^ Values reported for merged data sets (1.0 and 5.0 mg∙mL^–1^). ^c^ Dry volume from: *http://www.basic.northwestern.edu/biotools/proteincalc.html*

**SUPPLEMENTARY REFERENCES**

1. Andrade, S.L.A. & Einsle, O. The Amt/Mep/Rh family of ammonium transport proteins. *Molecular Membrane Biology* **24**, 357-365 (2007).

2. Willett, J.W. & Kirby, J.R. Genetic and Biochemical Dissection of a HisKA Domain Identifies Residues Required Exclusively for Kinase and Phosphatase Activities. *PLoS Genetics* **8**(2012).

3. Andrade, S.L.A., Dickmanns, A., Ficner, R. & Einsle, O. Crystal structure of the archaeal ammonium transporter Amt-1 from *Archaeoglobus fulgidus*. *Proceedings of the National Academy of Sciences of the United States of America* **102**, 14994-14999 (2005).

4. Marina, A., Waldburger, C.D. & Hendrickson, W.A. Structure of the entire cytoplasmic portion of a sensor histidine-kinase protein. *EMBO Journal* **24**, 4247-4259 (2005).

5. Buchan, D.W.A., Minneci, F., Nugent, T.C.O., Bryson, K. & Jones, D.T. Scalable web services for the PSIPRED Protein Analysis Workbench. *Nucleic Acids Research* **41**, W349-W357 (2013).

6. McWilliam, H. et al. Analysis Tool Web Services from the EMBL-EBI. *Nucleic Acids Research* **41**, W597-W600 (2013).

7. Wacker, T., Garcia-Celma, J.J., Lewe, P. & Andrade, S.L.A. Direct observation of electrogenic NH_4_^+^ transport in ammonium transport (Amt) proteins. *Proceedings of the National Academy of Sciences of the United States of America* **111**, 9995-10000 (2014).

8. Andrade, S.L. & Einsle, O. The Amt/Mep/Rh family of ammonium transport proteins. *Molecular membrane biology* **24**, 357-65 (2007).

9. Weiss, M. & Hilgenfeld, R. On the use of the merging *R* factor as a quality indicator for X-ray data. *Journal of Applied Crystallography* **30**, 203-205 (1997).

10. Karplus, P.A. & Diederichs, K. Linking Crystallographic Model and Data Quality. *Science* **336**, 1030-1033 (2012).
